# Mechanistic studies of DepR in regulating FK228 biosynthesis in *Chromobacterium violaceum* no. 968

**DOI:** 10.1371/journal.pone.0196173

**Published:** 2018-04-19

**Authors:** Yongjian Qiao, Tiantian Tong, Jiao Xue, Wenjing Lin, Zixin Deng, Yi-Qiang Cheng, Dongqing Zhu

**Affiliations:** 1 The Key Laboratory of Combinatorial Biosynthesis and Drug Discovery (Ministry of Education), Wuhan University, Wuhan, Hubei Province, China; 2 UNT System College of Pharmacy, University of North Texas Health Science Center, Fort Worth, Texas, United States of America; Universite Paris-Sud, FRANCE

## Abstract

DepR, a LysR-type transcriptional regulator encoded by the last gene of the putative *min* operon (*orf21-20-19-depR*) located at the downstream region of the anticancer agent FK228 biosynthetic gene cluster in *Chromobacterium violaceum* No. 968, positively regulates the biosynthesis of FK228. In this work, the mechanism underlining this positive regulation was probed by multiple approaches. Electrophoretic mobility shift assay (EMSA) and DNase I footprinting assay (DIFA) identified a conserved 35-nt DNA segment in the *orf21-orf22* intergenic region where the purified recombinant DepR binds to. Quantitative reverse transcription PCR (RT-qPCR) and green fluorescent protein (GFP) promoter probe assays established that transcription of phasin gene *orf22* increases in the *depR* deletion mutant of *C*. *violaceum* (CvΔdepR) compared to the wild-type strain. FK228 production in the *orf22*-overexpressed strain *C*. *violaceum* was reduced compared with the wild-type strain. DepR has two conserved cysteine residues C199 and C208 presumed to form a disulfide bridge upon sensing oxidative stress. C199X point mutations that locked DepR in a reduced conformation decreased the DNA-binding affinity of DepR; T232A or R278A mutation also had a negative impact on DNA binding of DepR. Complementation of CvΔdepR with any of those versions of *depR* carrying a single codon mutation was not able to restore FK228 production to the level of wild-type strain. All evidences collectively suggested that DepR positively regulates the biosynthesis of FK228 through indirect metabolic networking.

## Introduction

Bacterial cytokinesis is a complex process initiated by the formation of the Z ring, a dynamic structure formed of the tubulin homologue FtsZ. The *min* operon is one of the regulatory systems identified in *Escherichia coli* and most Gram negative bacteria, which prevents FtsZ polymerization near the cell poles [1–3]. The Min system comprises MinC, MinD and MinE proteins encoded by three genes organized as *minCDE* operon. The Min proteins blocks Z-ring assembly near the poles of the cell via the spatial regulation of the FtsZ polymerization inhibitor MinC, which has activity only when bound by MinD [4, 5]. MinE dissociates the MinCD complex at midcell [6]. In *Neisseria gonorrhoeae*, the *min* operon is composed of four genes, *minCDE* and a LysR-type transcriptional regulator (LTTR) gene *oxyR*. OxyR regulates the expression of *minD* [7].

**Fig 1:**
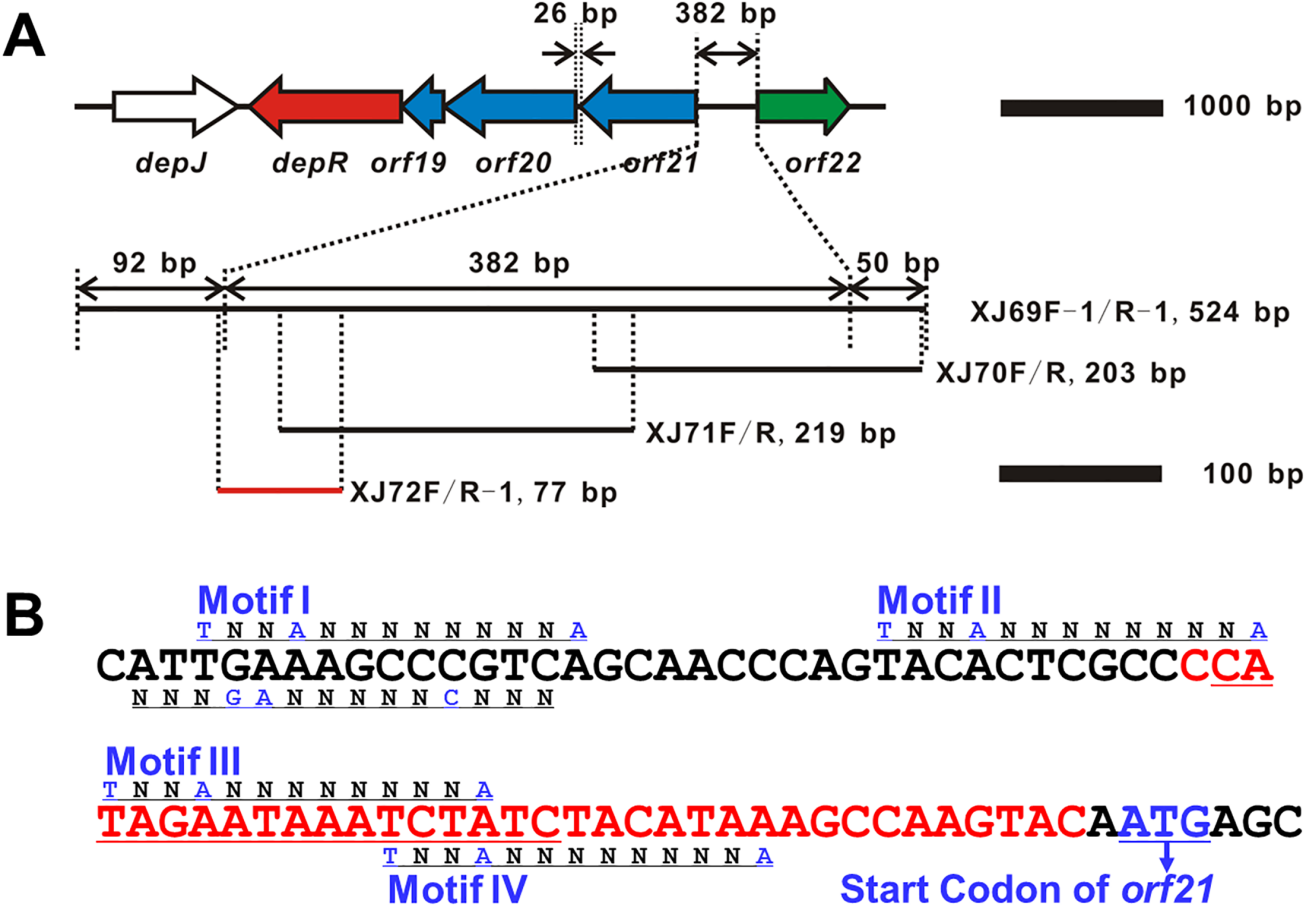
Genetic organization of the end of *dep* gene cluster and a conserved phasin gene (*orf22*) that encompasses a putative *min* operon (*orf21-20-19-depR*) and nucleotide sequence of DepR binding region. (A) Map of genes, intergenic regions, PCR primers and DNA fragments used in EMSAs. (B) Nucleotide sequence and motifs of a 77-bp DNA fragment to which DepR binds. Motif I-IV (TN_2_AN_8_A), predicted 13-bp LTTR consensus-binding motifs; N_3_GAN_5_CN_3_, predicted RcsB consensus-binding motif; red DNA region, the exact DepR-binding site defined by DIFA; red and underlined DNA region is consistent with the characterized OxyR-binding site in *E*. *coli*.

A similar organization of *minCDE-oxyR* operon was also found in *C*. *violaceum* No. 968 (Fig 1A), a producer of FK228, the most studied histone deacetylase inhibitor [8–11]. FK228 was approved by the FDA for the treatment of cutaneous T-cell lymphoma and peripheral T-cell lymphoma [12–14]. A 37-kb DNA region carrying a hybrid nonribosomal peptide synthetase-polyketide synthase (NRPS-PKS) gene cluster (*dep*) has been identified to be responsible for the biosynthesis of FK228 [15–18]. *depR* is located at the right boundary of the *dep* gene cluster and encodes a 312-aa protein with 60% identity and 76% similarity at amino acid sequence level to *N*. *gonorrhoeae* OxyR (306 aa). DepR is a positive regulator governing FK228 biosynthesis [17]. *depR* is peculiarly co-transcribed with three upstream genes *orf21-20-19*, which constitute the putative *min* operon and encode three Min proteins with 44%, 76%, and 59% identities and 61%, 86% and 81% similarities to MinC, MinD and MinE from *N*. *gonorrhoeae*, respectively. Deletion of *depR* severely reduced FK228 production but did not affect the transcriptional levels of the putative *min* genes [17].

In this study, we intended to delineate how DepR positively regulates FK228 biosynthesis through multiple approaches.

## Materials and methods

### Bacterial strains, plasmids, primers and general materials and methods

The bacterial strains and plasmids are listed in S1 Table. Primer sequences are listed in S2 Table.

Reagents and solvents were purchased from Sigma-Aldrich and were used without further purification. Restriction enzymes, T4 DNA ligase and DNA polymerase were purchased from New England BioLabs and used according to the manufacturer’s specifications. Ni-NTA affinity columns were purchased from GE Healthcare. DNA primers were synthesized by TsingKe Inc., Wuhan, China.

Growth media and conditions used for *E*. *coli* strains and standard methods for handling *E*. *coli in vivo* and *in vitro* were as described previously [19], unless otherwise noted. All DNA manipulations were performed following standard procedures [19]. DNA sequencing was carried out at TsingKe Inc. All proteins were handled at 4 °C unless otherwise stated. Protein concentrations were determined according to the method of Bradford, using a PerkinElmer Lambda 25 UV/Vis spectrophotometer with bovine serum albumin as standard [20]. Protein purity was estimated using SDS-PAGE and visualized using Coomassie Brilliant Blue stain. For genomic DNA preparation, *C*. *violaceum* was cultured in Luria-Bertani (LB) medium supplemented with 200 μg/mL ampicillin at 30 °C for 2 days [15]. The methods of *C*. *violaceum* transformation and conjugation were as described previously [15, 21].

### Expression and purification of recombinant DepR protein and DepR mutant proteins

A 939-bp DNA fragment harboring *depR* was obtained from plasmid pBMTL-3-*depR* digested with NdeI and HindIII and then ligated into the corresponding sites of pET-28a to give the expression plasmid pWHU1733 containing the *depR* gene fused to a His-tag coding sequence. Primers listed in S2 Table and a Fast Mutagenesis System kit (Transgen Biotech, Beijing, China) were used to introduce desired mutations into the *depR* gene on pWHU1733; mutant plasmids are listed in S1 Table. All mutations were confirmed by DNA sequencing.

The respective plasmids were introduced into *E*. *coli* BL21 (DE3) by transformation. The resulting recombinant *E*. *coli* transformant was cultured in LB medium containing 50 μg/mL kanamycin at 37 °C to an optical density at 600 nm of 0.6 to 0.8. After addition of 0.1 mM IPTG, the culture was incubated at 18 °C overnight. Cells were harvested by centrifugation and then resuspended in lysis buffer (50 mM Tris-HCl, 10% glycerol, 300 mM NaCl, 10mM imidazole, pH 7.5). After sonication, cell debris was removed by centrifugation at 20,000×*g* for 60 min and the supernatant was loaded onto a Ni-NTA column preequilibrated with lysis buffer. After the loaded column was washed with 20 mM imidazole in lysis buffer, proteins were eluted with elution buffer (50 mM Tris-HCl, 10% glycerol, 300 mM NaCl, 200 mM imidazole, pH 7.5). Elution fractions containing the target protein were identified by SDS-PAGE, pooled, concentrated and buffer-exchanged into storage buffer (50 mM NaH_2_PO_4_, 10% glycerol, pH 7.5) using a Biosharp Ultra Centrifugal filter (10 kD cut-off). The MW of protein His_6_-DepR was analyzed with a 5800 MALDI-TOF/TOF mass spectrometer (AB Sciex) by Sangon Biotech, Shanghai, China.

### Sequencing of the upstream region of *orf21*

The published DNA sequence of cosmid 18 (accession no. EF210776) carrying *dep* gene cluster contains a partial *orf21* sequence. Two pairs of primers XJ69F/XJ69R and YJ85F/YJ85R were used to amplify the missing region of *orf21* and its upstream sequence from the genomic DNA of *C*. *violaceum* No. 968. The PCR products were sequenced, and a 1341-bp DNA sequence was obtained (accession no. MG696726).

### Electrophoretic mobility shift assay (EMSA)

The binding site of DepR to the *orf21*-*orf22* intergenic region was determined by EMSA using the methods previously described [22–25]. Primers used to amplify putative target region DNA fragments are listed in S2 Table. Purified DepR was incubated with DNA fragments in a total volume of 20 μL at 30 °C for 30 min. The binding buffer contained 20 mM Tris-HCl (pH 8.0), 50 mM KCl, 10 mM MgCl_2_, 5% glycerol, and 0.5 mM EDTA. 0.5–1.5 mM DTT or H_2_O_2_ was added to the reaction mixture prior to incubation in order to change the redox status of DepR. The concentration of the dsDNA fragment was fixed at ca. 10 nM, while the concentrations of protein varied in the range of 0 to 30.7 μM. Incubated samples were applied to 2% agarose gel and electrophoresed on ice. Gels were stained with ethidium bromide and imaged with Syngene G:BOX F3 gel doc system.

### DNase I footprinting assay (DIFA)

A 130-bp promoter region of *orf21* was PCR amplified with the primer pair YJ39F/XJ72R-1, and cloned into pClone007 to generate pYJ01. DIFAs were performed similarly to the method of Wang *et al*. [26]. For preparation of fluorescent FAM labeled probes, the promoter region of *orf21* was amplified with primer pair M13F/M13R-48 (FAM) from pYJ01 using Dpx DNA polymerase (TOLO Biotech, Shanghai, China). The FAM-labeled PCR product, purified with a Wizard^®^ SV Gel and PCR Clean-Up System (Promega, USA), was quantified with NanoDrop 2000C spectrometer (Thermo, USA).

For each assay, 400 ng of DNA probe was incubated with different amounts of native DepR or mutated DepR(C199S) protein in a total volume of 40 μL at 25 °C for 30 min. A 10 μL solution containing 0.015 unit of DNase I (Promega, USA) and 100 nmol of freshly prepared CaCl_2_ was added to the reaction and further incubated at 25 °C for 1 min. Reaction was subsequently stopped by adding 140 μL of DNase I stop solution (200 mM unbuffered sodium acetate, 30 mM EDTA and 0.15% SDS) and extracted with phenol/chloroform, precipitated with ethanol, and redissolved in 30 μL of water. The preparation of DNA ladders, electrophoresis and data analysis were the same as described [26], except that the GeneScan-LIZ500 size standard (Applied BioSystems) was used.

### Determination of promoter activity by GFP production

The promoterless *gfp* gene from pWHU1421 (provided by Dr. L. Cheng, Wuhan University, unpublished) was used as reporter gene. *gfp* was amplified with primer pair DQ132F/DQ132R and cloned into the downstream NdeI/BglII sites of T7 promoter of pACYCDuet-1 to generate pWHU3026. The 388-bp intergenic region covering the promoter of *orf21* from *C*. *violaceum* was amplified using the primer pair XJ79F and XJ79R, inserted into the EcoNI+NdeI sites of pWHU3026 to replace the T7 promoter, and generated pWHU3027. The 388-bp intergenic region covering the promoter of *orf22* from *C*. *violaceum* was amplified using the primer pair 3T17F and 3T17R, inserted into the EcoNI+NdeI sites of pWHU3026 to replace the T7 promoter, and generated p3T11. The 525-bp intergenic region covering the promoter of *minC* from *E*. *coli* was amplified using the primer pair XJ80F and XJ80R, inserted into the EcoNI+NdeI sites of pWHU3026 to replace the T7 promoter, and generated pWHU3028. *E*. *coli* BL21(DE3) was transformed by introducing the plasmids, respectively.

The broad host-range gene expression vector pBMTL-3 was digested with BsaXI and XbaI, filled with Klenow fragment to create a blunt end, and ligated with T4 ligase to generate pYJ44 without a *lac* promoter region. The 1108-bp EcoRV/HindIII DNA fragment carrying the promoter region of *orf21* and *gfp* gene amplified from pWHU3027 with primer pair YJ81F-1/YJ80R, or the promoter region of *orf22* and *gfp* gene amplified from p3T11 with primer pair YJ82F-1/YJ80R, inserted into the corresponding sites of pYJ44 to generate pWHU3064 and pWHU3065, respectively. Each of the plasmids was transferred into *C*. *violaceum* wild-type strain or the *depR* mutant strain CvΔdepR using the method previously described [15, 21].

Promoter activity was measured as the rate of GFP production divided by the OD_600_ of the culture at each time point [27, 28]. *E*. *coli* BL21 (DE3) (pWHU3028) and the negative control strain *E*. *coli* BL21 (DE3) (pACYCDuet-1) were cultured in LB medium containing 25 μg/mL chloramphenicol at 37 °C overnight, respectively. 2% (v/v) of the overnight culture was transferred in 20 mL fresh LB medium and was grown at 37 °C for 12 hours. Samples were withdrawn every two hours. *E*. *coli* BL21 (DE3) (pWHU3027, pET28a), BL21 (DE3) (pWHU3027, pWHU1733), BL21 (DE3) (p3T11, pET28a), and BL21 (DE3) (p3T11, pWHU1733) and the negative control stains *E*. *coli* BL21 (DE3) (pACYCDuet-1, pET28a) and BL21 (DE3) (pACYCDuet-1, pWHU1733) were cultured in LB medium containing 25 μg/mL chloramphenicol and 50 μg/mL kanamycin at 37 °C overnight, respectively. 2% (v/v) of the overnight culture was transferred in 20 mL fresh LB medium and was grown at 37 °C to an optical density at 600 nm of ca. 0.6. After the addition of 0.1 mM IPTG, the culture was further incubated at 28 °C for 12 hours. Samples were withdrawn every two hours. *C*. *violaceum* No. 968 (pWHU3064), CvΔdepR (pWHU3064), No. 968 (pWHU3065), and CvΔdepR (pWHU3065), and the negative control strains *C*. *violaceum* No. 968 (pYJ44) and CvΔdepR (pYJ44) were cultured in the fermentation medium (1% Difco nutrient broth and 1% glucose) containing 25 μg/mL chloramphenicol and 100 μg/mL ampicillin at 28 °C overnight, respectively. 1% (v/v) of the overnight culture was transferred in 20 mL fresh fermentation medium containing 25 μg/mL chloramphenicol and 100 μg/mL ampicillin and was grown at 28 °C for 72 hours. Samples were withdrawn every 8 hours or 12 hours. NanoDrop 2000C Spectrophotometer (Thermo Scientific) was used to test the optical density at 600 nm of the samples. Infinite M200 Pro (Tecan) was used to detect fluorescence of GFP (excitation/emission wavelength, 485/520).

### RNA extraction and quantitative reverse transcriptase PCR (RT-qPCR)

*C*. *violaceum* wild-type and CvΔdepR were cultured in fermentation medium (1% Difco nutrient broth and 1% glucose) at 28 °C and cells were harvested at an OD_600_ of 0.5–0.6 in triplicate. Total bacterial RNA was isolated using a RNeasy Mini Kit (Qiagen) according to manufacturer’s instruction. RNase-free DNase (Promega, USA) was used to digest and remove DNA from RNA samples. Absence of residual DNA in RNA samples was verified by control incubations of the primer pair YJ71F and YJ71R, in which, the reverse transcriptase step was omitted did not give any PCR-amplified product of 16S rDNA. The typical PCR reaction included the initial denaturation step (94 °C, 5 min), 35 cycles of amplification (94 °C, 30 s; 60 °C, 30 s; 72°C, 30 s), and then a final incubation (72 °C, 5 min). The quality and concentration of RNA was determined by UV/Vis spectrophotometry and by gel electrophoresis. RT-qPCR was used to compare the expression levels of selected genes in *C*. *violaceum* wild-type and CvΔdepR, using primers listed in S2 Table. Specifically, RNA was transcribed into cDNA using EasyScript One-Step gDNA Removal and cDNA Synthesis SuperMix (Transgen) according to manufacturer’s instruction. qPCR was performed using AceQ qPCR SYBR Green Master Mix (Vazyme). A 7900HT Fast Real-Time PCR system (Applied BioSystems) was used to complete the reactions. The values were normalized to 16S rRNA expression for each sample. The expression levels of *orf19*, *orf21* and *orf22* in CvΔdepR were calculated relative to those of the wild-type strain using ΔΔ*C*_*T*_ [29].

### Detection and quantification of FK228 production by LC-MS

FK228 production was quantified similarly to the method previously described [15, 16]. Wild-type, mutant, and complementation strains of *C*. *violaceum* were cultured in 20 mL of fermentation medium with 5% (w/v) of Diaion HP-20 resins at 28 °C for 3 days. After fermentation cells and resins were collected together by centrifugation and lyophilized to dryness. Ten mL ethyl acetate was used to extract the dried mass and the organic extracts were concentrated on a rotovap. The residue was resuspended in 350 μL methanol and 15 μL of such organic extract was analyzed with a Thermo Scientific LTQ Orbitrap LC-MS for the detection and quantification of FK228 production.

## Results

### Identification of the DepR-binding site

The *depR* gene was excised from pBMTL-3-*depR* and cloned into pET28a for overexpression in *E*. *coli* BL21(DE3). Recombinant DepR carrying an N-terminal His_6_ tag (predicted, *m/z* 36,182.21 Da) was purified to >90% purity with Ni^2+^-NTA columns (S1A Fig), which showed a subunit *M*_D_ by MALDI-TOF/TOF MS of *m*/*z* 36,043 (predicted P-Met, 36,043.35). In addition, the presence of multiple MS signals at *m*/*z* 72,079, 108,120 and 144,301 suggested that DepR may be present in the solution as a dimer, trimer or tetramer (S2 Fig).

**Fig 2:**
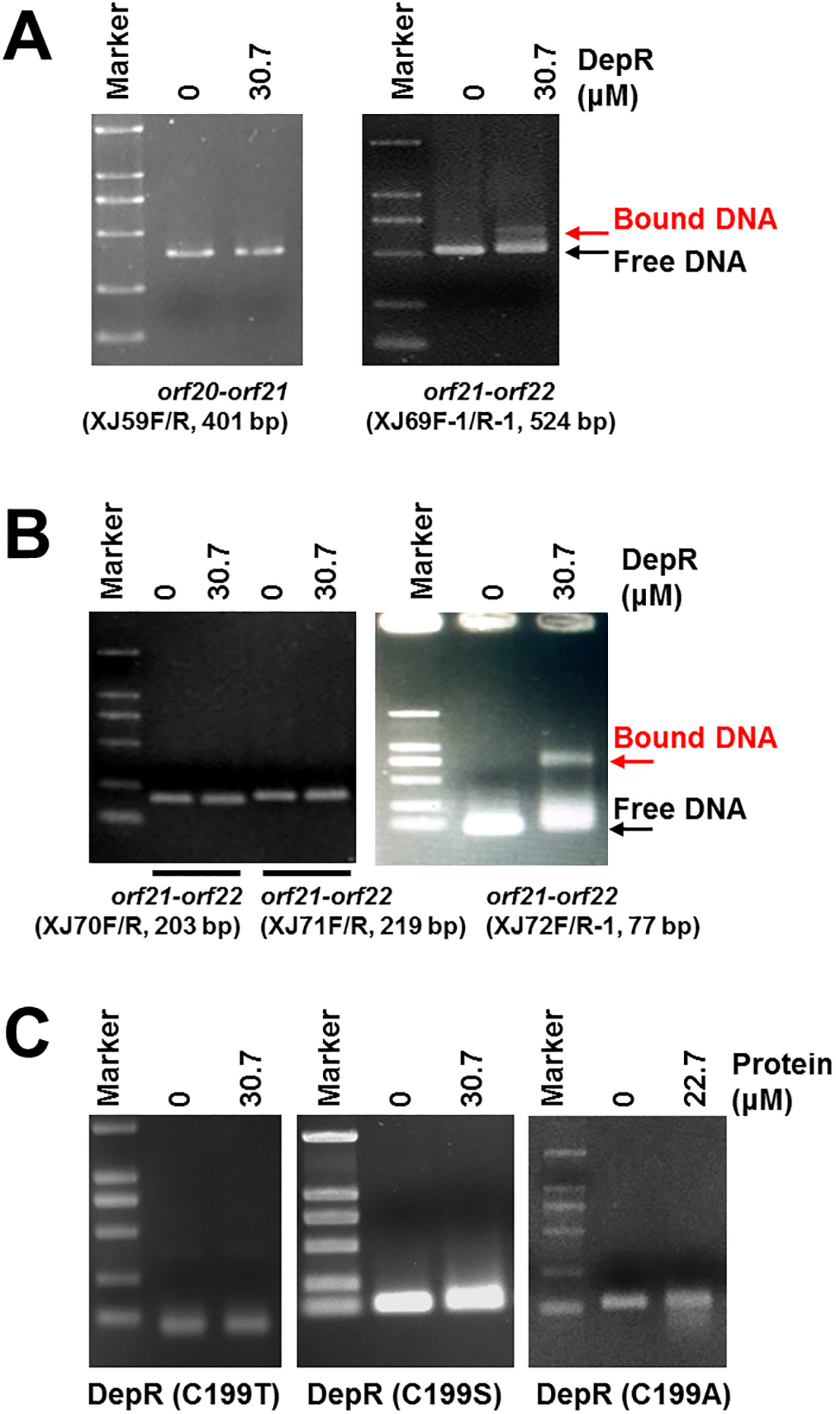
EMSAs of DepR with DNA fragments from the *orf22-21-20-19-depR* region. (A) EMSAs of DepR with DNA fragments harboring the intergenic region of *orf20-orf21* or the intergenic region of *orf21-orf22*. (B) EMSAs of DepR with DNA fragments covering different parts of the intergenic region of *orf21-orf22*. (C) EMSAs of three DepR mutants with target DNA fragment of DepR. Delete “Effects of DTT and H_2_O_2_ on the binding of DepR to target DNA fragments. (D)”.

The upstream region of *orf20* may contain a DepR-binding region based on the previous research on OxyR, the homologue of DepR in *N*. *gonorrheae* [7]. Initially a 401-bp DNA fragment carrying the 26-bp *orf20*-*orf21* intergenic region (Fig 1A) was obtained by PCR amplification using the primer pair XJ59F/XJ59R. However, EMSAs did not detect an obvious binding of the purified DepR to this stretch of DNA (Fig 2A, Left). Since *depR* as the last gene of the putative *min* operon is co-transcribed with *orf21*-20-*19* [17], the upstream region of *orf21*, the first gene of the putative *min* operon, may contain a DepR-binding region. The published DNA sequence of cosmid 18 (accession no. EF210776) carrying the *dep* gene cluster only contains a partial 3’-*orf21* sequence, so we amplified and sequenced a 1341-bp new DNA which contains a 392-bp 5’-*orf21*, a 567-bp ORF (named *orf22* encoding a putative phasin-family protein) and a 382-bp intergenic region *orf21-orf22* (S3 Fig). EMSAs using DepR and a 524-bp DNA fragment carrying the 382-bp *orf21*-*orf22* intergenic region, obtained by PCR amplification using the primer pair XJ69F-1/XJ69R-1, showed a protein-DNA binding (Fig 2A, Right). To further refine the actual DNA-binding target of DepR, primers were used to amplify different portions of the *orf21-orf22* intergenic region (Fig 1A and S2 Table), and the EMSA using DepR and a 77-bp DNA fragment obtained by PCR amplification using the primer pair XJ72F/XJ72R-1, showed a protein-DNA binding (Fig 2B, Right). EMSAs using DepR and a 203-bp or a 219-bp DNA fragment obtained by PCR amplification using the primer pair XJ70F/XJ70R or XJ71F/XJ71R did not show any detectable protein-DNA binding (Fig 2B, Left).

**Fig 3:**
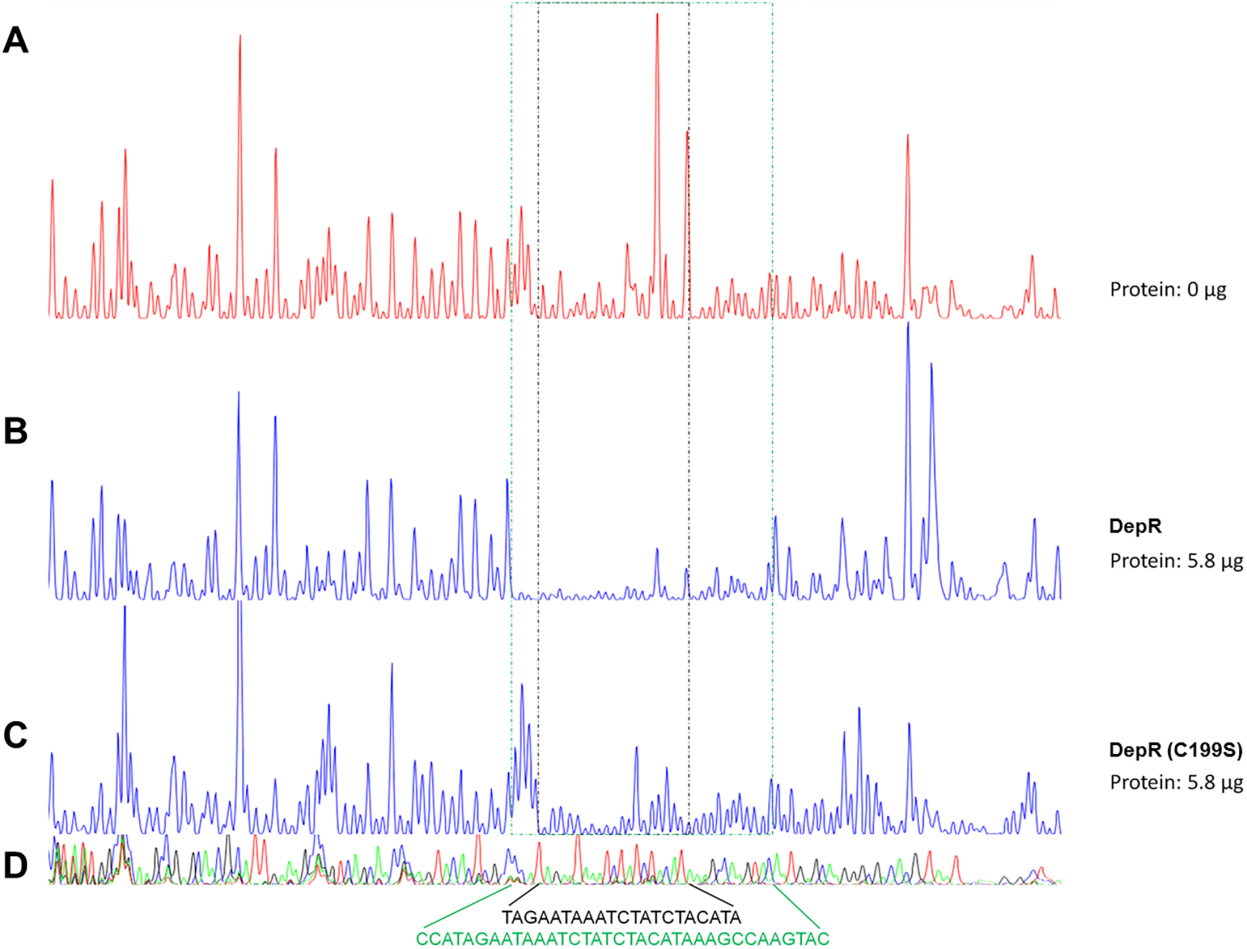
Identification of the exact DepR-binding sites in the *orf22*-*orf21* intergenic region using DIFAs. (A) The electropherogram of a reaction in the absence of protein. (B) The electropherogram of a reaction in the presence of 5.8 μg (4 μM) purified protein DepR. (C) The electropherogram of a reaction in the presence of 5.8 μg (4 μM) purified protein DepR (C199S). (D) The electropherogram of a sequencing reaction. The DNA sequences show DepR (green) or DepR (C199S) (black) binding sites.

This 77-bp DNA region upstream of the start codon of *orf21* contains four 13-bp LTTR consensus-binding motifs (T-N_11_-A) (Fig 1B, Motif I-IV). Alignment of these four 13-bp motifs showed the fourth nucleotide is also consensus (T-N_2_-A-N_8_-A). DIFAs in the presence of 4 μM DepR revealed that the exact DepR-binding site covered a 35-nt region (Fig 3), which is located at 36–2 nt upstream of the start codon of *orf21* (Fig 1B, red DNA region). This exact DepR-binding site harbors a 17-bp region (cATAGaaTAAATCTATC; the capital letters are consistent and the small letters are inconsistent.) (Fig 1B, red and underlined DNA region) that is also mostly consistent with the characterized OxyR-binding site (GATAGBYHWDRVCTATC) in *E*. *coli* [30].

### Repression of the expression of phasin protein-coding gene *orf22* by DepR

The previous semi-quantitative RT-PCR results did not suggest a regulatory role of DepR on the expression of the putative *min* operon in *C*. *violaceum* No. 968 [17]. To probe a possible regulatory role of DepR on the expression of *orf22*, green fluorescent protein (GFP) expression assays and RT-qPCR were performed.

**Fig 4:**
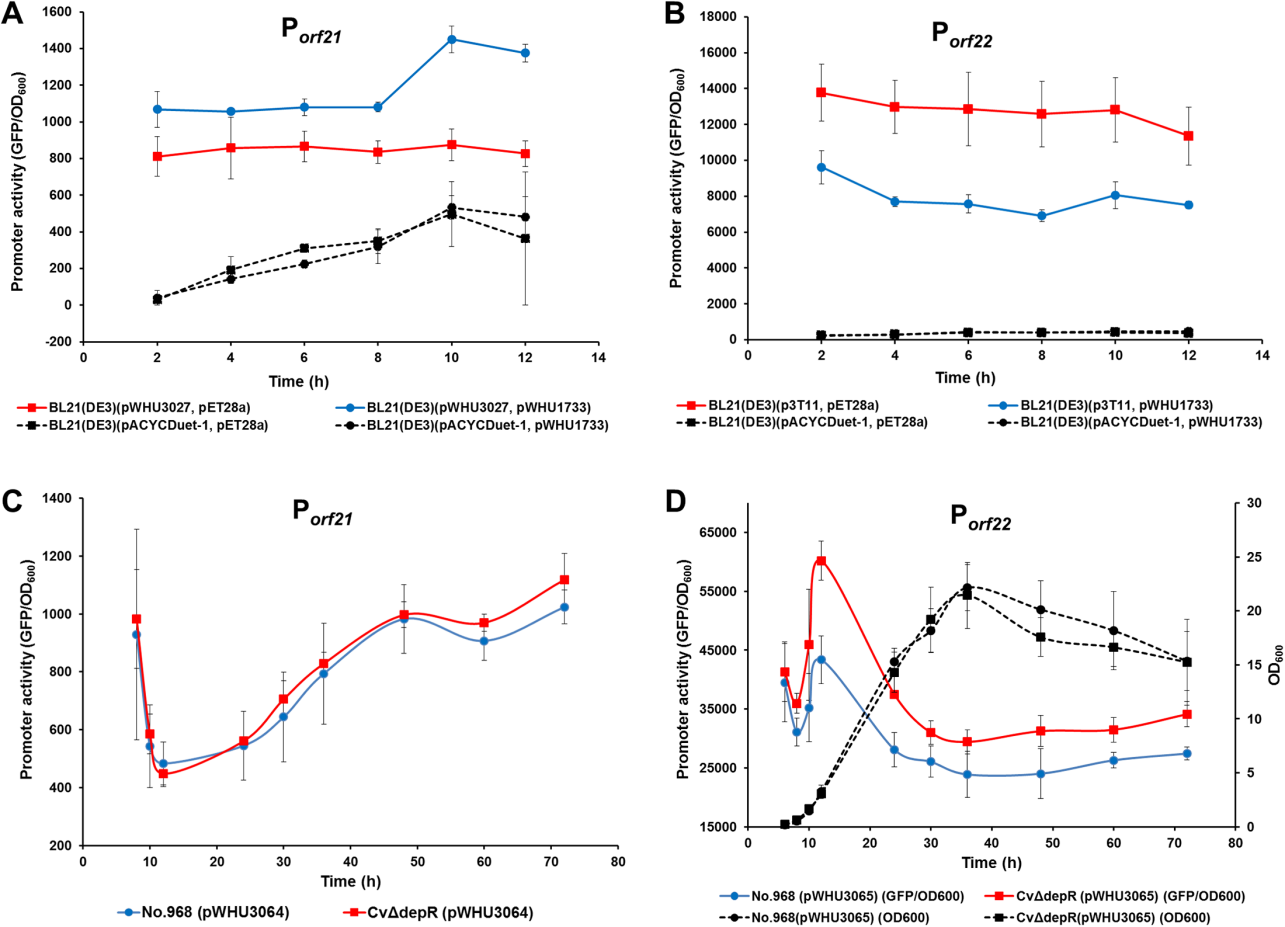
GFP expression assays of the promoter of *orf21* (P_*orf21*_) and the promoter of *orf22* (P_*orf21*_). (A) P_*orf21*_ in *E*. *coli* strain BL21(DE3); (B) P_*orf22*_ in *E*. *coli* strain BL21(DE3); (C) P_*orf21*_ in *C*. *violaceum* wild-type strain and CvΔdepR mutant strain; (D) P_*orf22*_ in *C*. *violaceum* wild-type strain and CvΔdepR mutant strain. Error bars indicate the standard deviation (n = 3).

GFP expression assays were performed in *E*. *coli* firstly, since the FK228 biosynthetic genes can be expressed in *E*. *coli* as a heterologous host [18]. Plasmid pWHU3026 carrying a *gfp* gene was constructed to be a promoter probe vector in *E*. *coli*. The promoter region of *minC* in *E*. *coli* was amplified and replaced the T7 promoter upstream of *gfp* in pWHU3026 to yield pWHU3028 as a positive control. GFP production in *E*. *coli* BL21(DE3) (pWHU3028) reached 600 to 900 GFP/OD_600_ throughout the 12-hour test period when it was under the control of *minC* promoter from *E*. *coli* (S4 Fig). Similarly, the *orf21-orf22* intergenic region was amplified by PCR and replaced the T7 promoter upstream of *gfp* in pWHU3026 in either direction to generate pWHU3027 (corresponding to the putative promoter of *orf21*, P_*orf21*_) or p3T11 (corresponding to the putative promoter of *orf22*, P_*orf22*_). Comparing to the negative controls of *E*. *coli* BL21(DE3) (pACYCDuet-1, pET28a) and BL21(DE3) (pACYCDuet-1, pWHU1733), the level of GFP under the control of P_*orf21*_ also reached ca. 800 GFP/OD_600_ [Fig 4A, BL21(DE3) (pWHU3027, pET28a)], and *depR* overexpression increased the GFP production ca. 23–66% [Fig 4A, BL21(DE3) (pWHU3027, pWHU1733)]. The level of GFP under the control of P_*orf22*_ reached ca. 12,000 GFP/OD_600_ [Fig 4B, BL21(DE3) (p3T11, pET28a)], significantly higher than the level of GFP promoted by P_*orf21*_. It is interesting to note that *depR* overexpression repressed the level of GFP under the control of P_*orf22*_ ca. 30–45% (Fig 4B, BL21(DE3) (p3T11, pWHU1733)). Those results indicated that DepR activates the *orf21* promoter and represses the *orf22* promoter in the heterologous host *E*. *coli*.

To perform GFP expression assays in *C*. *violaceum*, the *lac* promoter of pBMTL-3 was deleted to generate pYJ44, and two versions of the pYJ44-derived plasmids, pWHU3064 and pWHU3065 harboring *gfp* under the control of P_*orf21*_ or P_*orf22*_, were constructed and conjugated into No. 968 and CvΔdepR. The vector pYJ44 was also conjugated into these two strains as negative controls. Unlike the results in *E*. *coli*, GFP level under the control of P_*orf21*_ did not show a difference between in CvΔdepR and in the wild-type strain, which remained 400–1,400 GFP/OD_600_ during the testing period (Fig 4C). The level of GFP under the control of P_*orf22*_ in *C*. *violaceum* reached ca. 20,000–60,000 GFP/OD_600_ (Fig 4D), significantly higher one to two orders of magnitude than the level of GFP promoted by P_*orf21*_. GFP under the control of P_*orf22*_ in CvΔdepR was ca. 15–40% higher than that in the wild-type strain (Fig 4D). According to the growth curve of *C*. *violaceum*, the GFP level under the control of P_*orf22*_ increased and reached a maximum in the exponential phase following by a rapid decrease, and then kept relatively stable throughout the stationary phase and decline phase.

**Fig 5:**
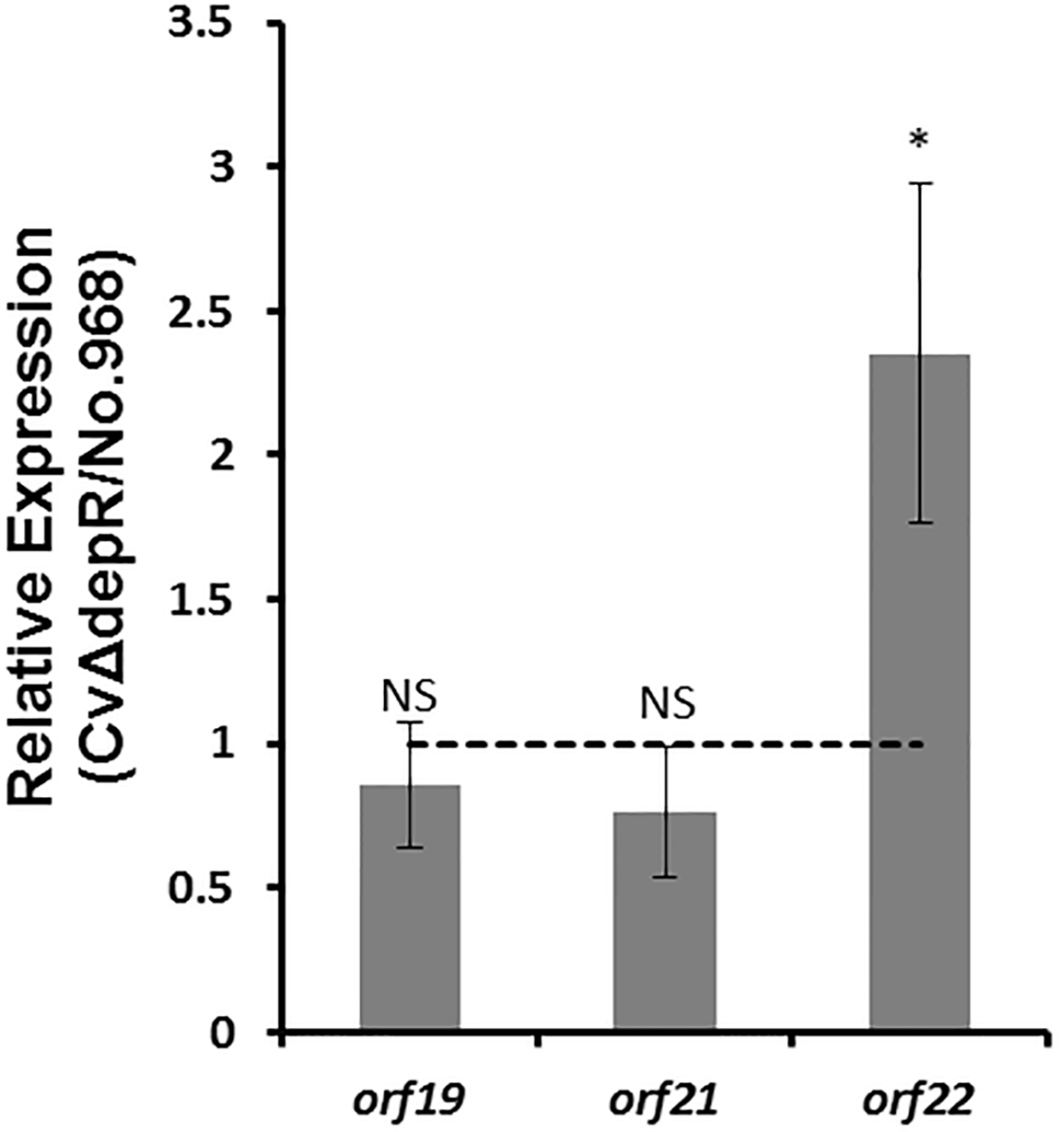
Effects of *depR* deletion on the expression of *orf19*, *orf21 and orf22*. Fold changes in the expression levels of *orf19*, *orf21* and *orf22* in the exponential phase of *depR* mutant strain CvΔdepR as compared to the wild-type strain. Using gene specific primers, RT-qPCR on total RNA from CvΔdepR and the wild-type strain. Data analysis was done as described in Materials and methods. Data are mean values of results from duplicate independent experiments with error bars indicating standard deviation. Statistical significance was determined by Student’s test. *, P<0.05; NS, not significant. Error bars indicate the standard deviation (n = 3).

To investigate the regulatory role of DepR by performing comparative transcriptional analysis between the wild-type and the CvΔdepR strain, RT-qPCR experiments were performed with RNA isolated from the wild-type and CvΔdepR strain grown in fermentation medium to an optical density at 600 nm of 0.5–0.6. The transcription levels of *orf19* and *orf21* in the putative *min* operon did not decrease significantly in CvΔdepR; in contrast, the transcription level of *orf22* increased ca. one fold in CvΔdepR (Fig 5). Those results indicated that DepR represses the *orf22* promoter in *C*. *violaceum*.

### Amino acid residues important for DepR binding

OxyR, a LysR-type transcriptional regulator with two conserved cysteine residues C199 and C208, was the first discovered redox-sensitive factor that is activated by a thiol-disulfide switch [31–35]. OxyR alters its contact with DNA under oxidizing or reducing conditions. The redox status of OxyR can be manipulated by adding DTT as a reductive agent or H_2_O_2_ as an oxidative one. An OxyR (C199S) mutant locks in the reduced status, which behaves like reduced OxyR generated by using DTT [36]. The T238 residue of OxyR is buried in the core of the region containing the redox-active C199 and C208. A T238A mutation might impair the disulfide bond formation and lock the mutated protein in a reduced status [37]. D142 and R273 may locate the region of OxyR that interacts with RNA polymerase. OxyR (D142A) and OxyR (R273H) mutants no longer activate the expression of its target gene but only partially lose their DNA binding capacity [37]. On the basis of the extensively studied OxyR, we did EMSAs using purified DepR under oxidizing or reducing conditions. Sequence alignment of DepR and OxyR (GenBank, WP_001025939) was used aid in point mutants of DepR. We prepared six DepR mutants in which the selected residues correspond to the above-mentioned amino acids in OxyR. EMSAs of wild-type DepR and the DepR target DNA fragment were used as positive controls in the following EMSAs of DepR mutants.

In EMSA using native DepR and the 130-bp DNA fragment carrying the DepR target region, addition of 0.5–1.5 mM DTT resulted in less DNA binding; the bound DNA band began to disappear as the concentration of DTT increased (S6A Fig). H_2_O_2_ added at 0.5–1.5 mM had little or no effect on DepR-DNA binding (S6B Fig).

To precisely define the residue(s) undermining DepR-DNA binding, three point-mutated proteins, DepR (C199T), DepR (C199S) and DepR (C199A), were purified (S1A Fig), since the attack on C208 by C199 generates an intramolecular disulfide bond that drives massive conformational changes in the protein. EMSAs using the 77-bp or a 130-bp DNA fragment and those DepR (C199X) mutants showed that the bands of protein-bound DNA disappeared (Fig 2C). We carried out DIFA in the presence of 5.8 μg (4 μM) or 9.8 μg (6.8 μM) purified protein DepR (C199S) (Fig 3C, S5 Fig). The results revealed that the exact DepR(C199S)-binding site covered a 21-nt region located 33–13 nt upstream of the start codon of *orf21* (Fig 3D, the black DNA sequences). DepR (T244A) mutant was also generated and purified (S1B Fig). EMSA using DepR (T244A) and the 130-bp DNA fragment carrying the DepR target region showed that the bound DNA band was weakened compared with the positive control DepR (S7 Fig). DepR (D142A) was produced as insoluble inclusion bodies (not shown); DepR (R278A) was produced in soluble form and purified (S1B Fig). EMSA using the 130-bp DNA fragment carrying the 35-bp DepR target region according to the above DIFAs showed that DepR (R278A) lacked a DNA binding capacity (S7 Fig).

We also substituted three other conserved residues S229, T232 and R271 with alanine. DepR (R271A) was insoluble (not shown); DepR (S229A) and DepR (T232A) were soluble and purified (S1B Fig). EMSA showed that DepR (T232A) is defective in DNA binding (S7 Fig), while EMSA using DepR (S229A) and the 130-bp DNA fragment carrying the DepR target region or a 136-bp DNA fragment as negative control exhibited unexplainable smears.

In summary, the DNA binding ability for the deduced DepR decreased compared with the purified native DepR. The T232A or R278A mutation had a negative impact on DNA binding of DepR.

### Effects of point-mutations of DepR on FK228 production

**Fig 6:**
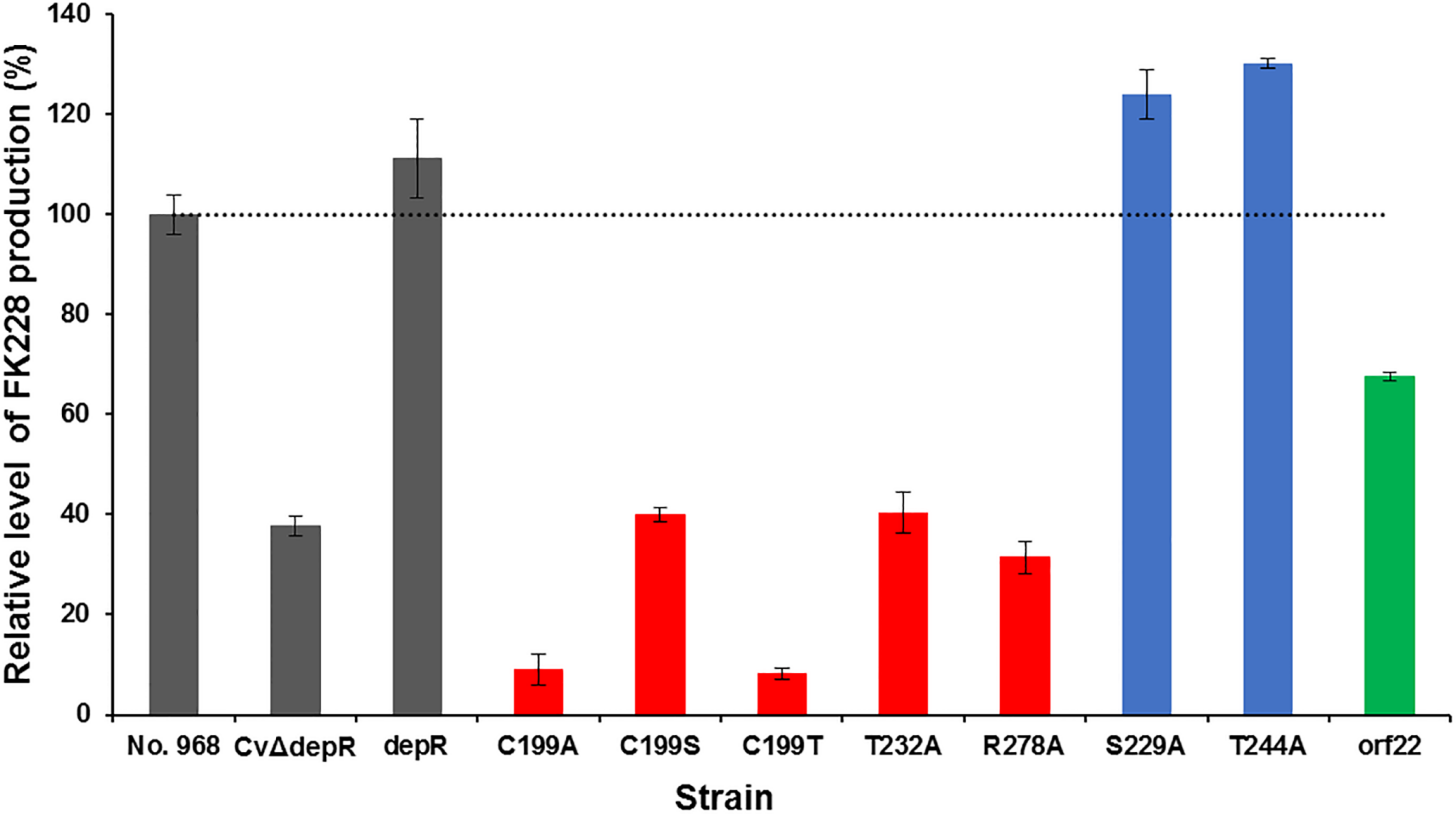
Relative levels of FK228 productions by *C*. *violaceum* strains detected and quantified by LC-MS. No. 968, the wild-type strain; CvΔdepR, *depR* gene deletion mutant strain. Eight complementation strains: *depR*, CvΔdepR (pBMTL-3-*depR)*; C199A, CvΔdepR (pWHU3057); C199S, CvΔdepR (p3T9); C199T, CvΔdepR (pWHU3058); T232A, CvΔdepR (pWHU3060); R278A, CvΔdepR (pWHU3062); S299A, CvΔdepR (pWHU3059); T244A, CvΔdepR (pWHU3061). An overproduction strain of *orf22*, No. 968 (pWHU3070). Data are mean values of results from duplicate experiments with error bars indicating standard deviation (n = 3).

Production of FK228 by CvΔdepR is severely impaired, while complementation of *depR* restores FK228 production [17]. To delineate the effects of point-mutations of DepR on FK228 production, seven *depR* point-mutated gene variants [*depR* (C199A), *depR* (C199S), *depR* (C199T), *depR* (S229A), *depR* (T232A), *depR* (T244A), *depR* (R278A)], each encoding a soluble mutated protein in *E*. *coli* protein expression system, were cloned into the expression vector pBMTL-3, respectively. The pBMTL-3-based plasmids were introduced into CvΔdepR to generate seven complementation strains (S1 Table), followed by fermentation. FK228 produced by each strain, including CvΔdepR as a negative control and the wild-type No. 968 strain and the CvΔdepR/pBMTL-3-*depR* complementation strain as positive controls, was quantified with LC-MS (Fig 6). Full FK228 production was not restored by complementation of CvΔdepR with *depR* (C199X), *depR* (T232A) or *depR* (R278A) (Fig 6, red), which encodes DepR mutant lacking a DNA binding capacity according to EMSAs (Fig 2 and 2D; S7 Fig). FK228 production was restored to full level or above by complementation of CvΔdepR with *depR* (S229A) or *depR* (T244A) (Fig 6, blue). These findings suggest that the reduced DepR cannot regulate the biosynthesis of FK228 positively and that the T232A or R278A mutation has a negative impact on the activation of DepR on FK228 production.

## Discussion

### Positive but indirect regulation of FK228 Biosynthesis by DepR

The previous experiments revealed that FK228 production and the transcription level of most *dep* genes, including *depABCDEFGH* and *depI*, decreased in the CvΔdepR mutant, which led to the conclusion that FK228 biosynthesis is positively regulated by DepR in *C*. *violaceum* No. 968 [17]. According to the operon organization of *dep* gene cluster, five intergenic regions (*depL-depM*, *depM-“depN”*, “*depN”-depA*, *depH-depI*, and *depI-depJ*) and an intragenic region (“*depN”*, a pseudogene upstream of *depA*) (S8A Fig) were amplified and used in EMSAs with purified DepR. Surprisingly, EMSAs did not show any detectable protein-DNA binding (S8B Fig). Two DNA fragments from the intragenic regions of *depM* and *depA*, respectively, were amplified and tested, but no protein-DNA binding was observed in EMSAs as well (S8B Fig). Furthermore, EMSAs with reduced DepR (in the presence of DTT) also does not show any detectable protein-DNA binding (not shown). These EMSAs suggested that the FK228 biosynthetic gene cluster doesn’t contain DepR-binding site.

Thailandepsins and spiruchostatins belong to the FK228-family of natural products, and their biosynthetic gene clusters (*tdp* and *spi*) have been characterized in *Burkholderia thailandensis* E264 and *Pseudomonas* sp. Q71576, respectively [38–40]. Unlike *depR*, which is located downstream of the *dep* gene cluster and encodes an OxyR-family transcriptional regulator, an AraC-family transcriptional regulator gene *tdpR* and a LysR-family transcriptional regulator gene *spiR* are located upstream of the *tdp* and *spi* gene clusters, respectively. TdpR and SpiR are not homologues of DepR according to their protein sequences. SpiR positively regulates the biosynthesis of spiruchostatin. The heterologous overexpression of DepR enhanced spiruchostatin production possibly through upregulating *spiR* expression [39]. All those results suggest that the positive regulatory function of DepR on the biosynthesis of FK228 may be indirect.

### Regulation of Min system and biosynthesis of polyhydroxyalkanoates (PHAs) by DepR

**Fig 7:**
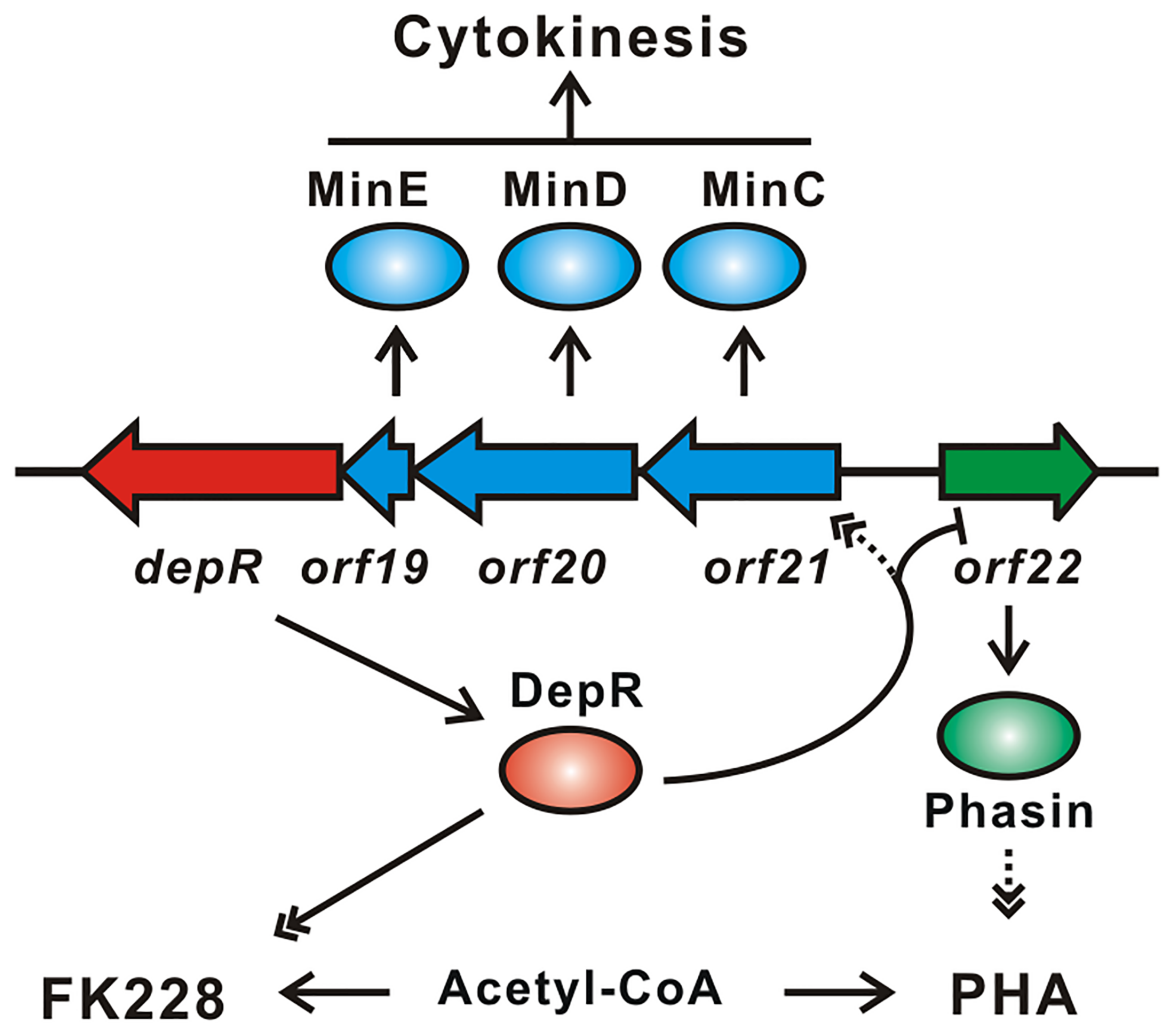
A model of regulation of cytokinesis, FK228 biosynthesis and PHA biosynthesis by DepR. The diagrams are defined as follows: arrow shapes, genes; ovals, proteins; lines with single arrow, biochemical reactions or life process; lines with double arrowheads, positive regulation; lines with a short bar, negative regulation; Dotted lines are predicted relationships without direct experimental data in *C*. *violaceum*.

Polyhydroxyalkanoates (PHAs) are polyesters produced by numerous bacteria as carbon and energy storage compounds [41]. Phasins, PHA granule-associated proteins, promote PHA biosynthesis and affect the number and size of PHA granules [42]. Some *C*. *violaceum* strains are known to produce PHAs [43–46]. The observed repression of the expression of *orf22* encoding a phasin protein by DepR prompted us to proposed that DepR regulates PHA biosynthesis though phasin protein Orf22. The Min system of cytokinesis is negatively related to PHA biosynthesis though DepR, since *depR* is the last gene of the putative *min* operon in *C*. *violaceum* (Fig 7).

GFP expression assays of P_*orf21*_ in the heterologous host *E*. *coli* suggested that DepR activates the putative *min* operon (Fig 4A), while the same result was not observed in the native host *C*. *violaceum* (Figs 4C and 5). This inconsistence may be due to the following one or two reasons. Firstly, the overexpression of *depR* in *E*. *coli* could have magnified or created an artificial regulatory effect of DepR. Secondly, there may be another layer of regulation of the putative *min* operon in the native host *C*. *violaceum*, which is absent in the heterologous host *E*. *coli*. In *Proteus mirabilis*, a member of the family Enterobacteriaceae, the transcription of *minCDE* is positively regulated by RcsB, the response regulator of the Rcs phosphorelay two-component signal transduction system [47]. The DepR-binding site revealed in this study does not include the first LTTR-binding motif (Fig 1B, Motif I) that is conserved in the intergenic region upstream of *minC* in many other bacterial species. Motif I overlaps a 14-bp region (ATTGAAAGCCCGTC) which is consistent with the characterized RcsB-binding boxes (N_3_-GA-N_5_-C-N_3_) in *E*. *coli* and *P*. *mirabilis* [48]. An RcsB homolog may regulate the transcription of the putative *min* operon in *C*. *violaceum* and interacts with DepR.

Since the transcription level of the putative *min* operon is not different in the wild-type No. 968 strain and the CvΔdepR mutant strain, we further probed the phasin protein gene *orf22*. Previous studies indicated that phasin proteins have an enhancing effect on PHA biosynthesis [49–53]. Gene *orf22* was inserted into pBMTL-3 to generate pWHU3070, which was transformed into No. 968, followed by fermentation and LC-MS. FK228 production in the *orf22*-overexpressed strain *C*. *violaceum* No.968 (pWHU3070) decreased by ca. 33% comparing to No. 968 (Fig 6, *orf22*). This observation promoted us to hypothesize that the enhancement of PHA biosynthesis induced by *orf22* overexpression may be one of reasons for a reduced production of FK228 in the CvΔdepR mutant. Because both FK228 biosynthesis and PHA biosynthesis utilize acetyl-CoA derivatives as building blocks, likely the two biosynthetic pathways have a competitive relationship for substrates (Fig 7).

Taken together, a possible model for gene regulation by DepR is shown in Fig 7, *depR* is co-transcribed with the putative *min* genes. DepR binds the intergenic region of the putative *minC* gene *orf21* and the putative phasin gene *orf22*, resulting in repression of *orf22* and activation of *orf21-20-19-depR*. As the phasin protein Orf22 reduces, PHA biosynthesis is downregulated and higher flux of acetyl-CoA derivatives is diverted towards FK228 biosynthesis. We also propose that DepR upregulates the transcription of FK228 biosynthetic genes through some other uncharacterized approaches.

## Supporting information

S1 Table Bacterial strains and plasmids used in this study. (DOC)

S2 Table Primers used in this study. (DOC)

S1 Fig SDS-PAGE analysis of recombinant His_6_-DepR and His_6_-DepR mutants. (TIF)

S2 Fig MALDI-TOF/TOF MS of recombinant His_6_-DepR. (TIF)

S3 Fig DNA sequence of gene *orf21*, gene *orf22*, and the *orf21-orf22* intergenic region. The reverse complementary sequence of *orf21* is marked in pink (previously published) and blue (determined in this work). The *orf22* sequence (determined in this work) is marked in green. The start codons and stop codons of *orf21* and *orf22* are indicated by underlining. The *orf21-orf22* intergenic region is marked in red. The 77-bp DNA region bound by DepR is shown in bold. The sequence has been deposited in GenBank with Accession Number MG696726.
(PDF)

S4 Fig GFP expression assays of the *minC* promoter of *E*. *coli* strain BL21(DE3). (TIF)

S5 Fig Identification of the DepR(C199S)-binding site in the *orf22-orf21* intergenic region using DIFA. The upper two electropherograms indicate the reactions in the absence of protein and the presence of 9.8 μg (6.8 μM) purified protein DepR(C199S). The lower electropherogram represents the sequencing reaction. The DNA sequence (black) shows DepR (C199S) binding sites.
(TIF)

S6 Fig Effects of DTT and H_2_O_2_ on the binding of DepR to target DNA fragments. (A) EMSA analysis of DepR with target DNA fragments of DepR and 0–1.5 mM DTT. (B) EMSA analysis of DepR with target DNA fragments of DepR and 0–1.5 mM H_2_O_2_.
(TIF)

S7 Fig EMSA analysis of DepR mutants with target DNA fragments of DepR. The bound DNA bands were indicated by yellow arrows.
(TIF)

S8 Fig EMSA analysis of DepR with DNA fragments from *dep* gene cluster. (A) *dep* gene cluster, primer pairs and the sizes and locations of DNA fragments amplified using in EMSA. (B) EMSA analysis of DepR with DNA fragments.
(TIF)
