# Supplementary material for: Mechanistic studies of DepR in regulating FK228 biosynthesis in Chromobacterium violaceum no. 968
Source: PLoS One. 2018 Apr 19;13(4):e0196173. doi: 10.1371/journal.pone.0196173 (PMC5908139; doi:10.1371/journal.pone.0196173)
Supplement: S1 Table — (DOC) [file pone.0196173.s001.doc]

**S1 Table.**

| Strain or plasmid | | Relevant phenotype and/or characteristics | Source or reference |
| --- | --- | --- | --- |
| *Chromobacterium violaceum* | | |  |
| No. 968 | Wild-type strain, FK228 producer | |  |
| CvΔdepR | Gene *depR* deletion mutant strain | |  |
| CvΔdepR/pBMTL-3-*depR* | Complementation of CvΔdepR with *depR* carried on pBMTL-3 | |  |
|  |  | |  |
| *Escherichia coli* |  | |  |
| DH10B | F^-^ *mcrA, Δ(mrr-hsdRMS-mcrBC),* φ80d*lacZΔM15, ΔlacX74, recA1, endA1, araD139, Δ(ara, leu)7697, galU, galK, rpsL, nupG* | | Gibco BRL |
| BL21 (DE3) | F^–^ *ompT* *hsdS*_B_ (r_B_^-^m_B_^-^) *gal* *dcm* (DE3) | | Invitrogen |
|  |  | |  |
| Plasmids |  | |  |
| Cosmid 18 | Amp^r^, Kan^r^, cosmid clone containing the FK228 biosynthetic gene cluster | |  |
| pACYCDuet-1 | Cml^r^, *ori*^P15A^, *lacI*, T7 promoter | | Novagen |
| pBMTL-3 | Cml^r^, broad host-range gene expression vector | |  |
| pBMTL-3-*depR* | Cml^r^, *depR* (with RBS from pET29a) cloned into pBMTL-3 | |  |
| pClone007 | Amp^r^, *ori*^pUC^ | | TsingKe |
| pET28a | Kan^r^, *ori*^f1^, *lacI*, *ori*^pBR322^, T7 promoter | | Novagen |
| pWHU1421 | Promoterless *gfpmut3* gene cloned into pMD18-T | | L. Cheng, unpublished |
| p3T9 | pBMTL-3-*depR* (C199S): 939-bp NdeI + HindIII DNA fragment harboring *depR* (C199S) released from pWHU3033, inserted into the corresponding sites of pBMTL-3-*depR* to replace the wild-type *depR* | | This work |
| p3T11 | pACYCDuet-1-P*_orf22_*-*gfp*: 388-bp EcoNI + NdeI DNA fragment carrying the promoter region of *orf22* amplified from the DNA of *C. violaceum* No. 968 by using primer pair 3T17F and 3T17R, inserted into EcoNI + NdeI site of pWHU3026 | | This work |
| pWHU1733 | pET28a-*depR*: 939-bp NdeI + HindIII DNA fragment carrying *depR* recovered from pBMTL-3-*depR*, inserted into NdeI + HindIII site of pET28a | | This work |
| pWHU3026 | pACYCDuet-1-*gfp*: 738-bp NdeI + BglII DNA fragment carrying *gfp* amplified from p3T7 by using primer pair DQ132F and DQ132R, inserted into NdeI + BglII site of pACYCDuet-1 | | This work |
| pWHU3027 | pACYCDuet-1-P*_orf21_*-*gfp*: 388-bp EcoNI + NdeI DNA fragment carrying the promoter region of *orf21* amplified from the DNA of *C. violaceum* No. 968 by using primer pair XJ79F and XJ79R, inserted into EcoNI + NdeI site of pWHU3026 | | This work |
| pWHU3028 | pACYCDuet-1-P*_minC-Eco_*-*gfp*: 525-bp EcoNI + NdeI DNA fragment carrying the promoter region of *minC* amplified from the DNA of *E. coli* by using primer pair XJ80F and XJ80R, inserted into EcoNI + NdeI site of pWHU3026 | | This work |
| pWHU3032 | pET28a-*depR* (D142A): a pET28a-based plasmid harboring *depR* with a D142A mutation generated through site-directed mutagenesis using primer pair 3T9F and 3T9R to amplify the gene from pWHU1733 | | This work |
| pWHU3033 | pET28a-*depR* (C199S): a pET28a-based plasmid harboring *depR* with a C199S mutation generated through site-directed mutagenesis using primer pair 3T10F and 3T10R to amplify the gene from pWHU1733 | | This work |
| pWHU3034 | pET28a-*depR* (C199A): a pET28a-based plasmid harboring *depR* a C199A mutation generated through site-directed mutagenesis using primer pair 3T11F and 3T11R to amplify the gene from pWHU1733 | | This work |
| pWHU3035 | pET28a-*depR* (C199T): a pET28a-based plasmid harboring *depR* with a C199T mutation generated through site-directed mutagenesis using primer pair YJ7F and YJ7R to amplify the gene from pWHU1733 | | This work |
| pWHU3036 | pET28a-*depR* (S229A): a pET28a-based plasmid harboring *depR* with a S229A mutation generated through site-directed mutagenesis using primer pair 3T12F and 3T12R to amplify the gene from pWHU1733 | | This work |
| pWHU3037 | pET28a-*depR* (T232A): a pET28a-based plasmid harboring *depR* with a T232A mutation generated through site-directed mutagenesis using primer pair 3T13F and 3T13R to amplify the gene from pWHU1733 | | This work |
| pWHU3038 | pET28a-*depR* (T244A): a pET28a-based plasmid harboring *depR* with a T244A mutation generated through site-directed mutagenesis using primer pair 3T13F and 3T13R to amplify the gene from pWHU1733 | | This work |
| pWHU3039 | pET28a-*depR* (R271A): a pET28a-based plasmid harboring *depR* with a R271A mutation generated through site-directed mutagenesis using primer pair 3T14F and 3T14R to amplify the gene from pWHU1733 | | This work |
| pWHU3040 | pET28a-*depR* (R278A): a pET28a-based plasmid harboring *depR* with a R278A mutation generated through site-directed mutagenesis using primer pair 3T15F and 3T15R to amplify the gene from pWHU1733 | | This work |
| pWHU3057 | pBMTL-3-*depR* (C199A): 939-bp NdeI + HindIII DNA fragment harboring *depR* (C199A) released from pWHU3034, inserted into the corresponding sites of pBMTL-3-*depR* to replace the wild-type *depR* | | This work |
| pWHU3058 | pBMTL-3-*depR* (C199T): 939-bp NdeI + HindIII DNA fragment harboring *depR* (C199T) released from pWHU3035, inserted into the corresponding sites of pBMTL-3-*depR* to replace the wild-type *depR* | | This work |
| pWHU3059 | pBMTL-3-*depR* (S229A): 939-bp NdeI + HindIII DNA fragment harboring *depR* (S229A) released from pWHU3036, inserted into the corresponding sites of pBMTL-3-*depR* to replace the wild-type *depR* | | This work |
| pWHU3060 | pBMTL-3-*depR* (T232A): 939-bp NdeI + HindIII DNA fragment harboring *depR* (T232A) released from pWHU3037, inserted into the corresponding sites of pBMTL-3-*depR* to replace the wild-type *depR* | | This work |
| pWHU3061 | pBMTL-3-*depR* (T244A): 939-bp NdeI + HindIII DNA fragment harboring *depR* (T244A) released from pWHU3038, inserted into the corresponding sites of pBMTL-3-*depR* to replace the wild-type *depR* | | This work |
| pWHU3062 | pBMTL-3-*depR* (R278A): 939-bp NdeI + HindIII DNA fragment harboring *depR* (R278A) released from pWHU3040, inserted into the corresponding sites of pBMTL-3-*depR* to replace the wild-type *depR* | | This work |
| pWHU3064 | pYJ44-P*_orf21_*-*gfp*: 1108-bp EcoRV + HindIII DNA fragment carrying the promoter region of *orf21* and *gfp* amplified from pWHU3027 using primer pair YJ81F-1 and YJ80R, inserted into the corresponding sites of pYJ44 | | This work |
| pWHU3065 | pYJ44-P*_orf22_*-*gfp*: 1108-bp EcoRV + HindIII DNA fragment carrying the promoter region of *orf22* and *gfp* amplified from p3T11 using primer pair YJ82F-1 and YJ80R, inserted into the corresponding sites of pYJ44 | | This work |
| pWHU3070 | pBMTL-3-*orf22*: 567-bp NdeI + HindIII DNA fragment carrying gene *orf22* amplified from the DNA of *C. violaceum* No. 968 using primer pair YJ85F and YJ85R, inserted into the corresponding sites of pBMTL-3 | | This work |
| pYJ01 | 130-bp promoter region of *orf21* was PCR amplified with primer pair YJ39F and XJ72R-1, and inserted into the pClone007 vector | | This work |
| pYJ44 | pBMTL-3 digested with BsaXI and XbaI , filled with Klenow fragment to create a blunt end, and ligated with T4 ligase to generate a plasmid without the *lac* promoter region | | This work |

**References**

1. Cheng YQ, Yang M, Matter AM (2007) Characterization of a gene cluster responsible for the biosynthesis of anticancer agent FK228 in Chromobacterium violaceum No. 968. Appl Environ Microbiol 73: 3460-3469.

2. Potharla VY, Wesener SR, Cheng YQ (2011) New insights into the genetic organization of the FK228 biosynthetic gene cluster in Chromobacterium violaceum no. 968. Appl Environ Microbiol 77: 1508-1511.

3. Wesener SR, Potharla VY, Cheng YQ (2011) Reconstitution of the FK228 biosynthetic pathway reveals cross talk between modular polyketide synthases and fatty acid synthase. Appl Environ Microbiol 77: 1501-1507.

4. Lynch MD, Gill RT (2006) Broad host range vectors for stable genomic library construction. Biotechnol Bioeng 94: 151-158.
