# Supplementary material for: Mechanistic studies of DepR in regulating FK228 biosynthesis in Chromobacterium violaceum no. 968
Source: PLoS One. 2018 Apr 19;13(4):e0196173. doi: 10.1371/journal.pone.0196173 (PMC5908139; doi:10.1371/journal.pone.0196173)
Supplement: S2 Table — (DOC) [file pone.0196173.s002.doc]

**S2 Table.**

| Primer name | Sequence (5’-3’)*,  (Restriction enzyme sites are underlined) | Purpose |
| --- | --- | --- |
| 3T9F | CAAGCGGGGCGAGGTGgcgGCCATCATCGT | pET28a-*depR* (D142A) |
| 3T9R | cgcCACCTCGCCCCGCTTGAGCATTTCCG | pET28a-*depR* (D142A) |
| 3T10F | GCTGACCCAGGGCAATaGCTTCCGCGACCA | pET28a-*depR* (C199S) |
| 3T10R | GCtATTGCCCTGGGTCAGCAGTAAGACGCT | pET28a-*depR* (C199S) |
| 3T11F | GCTGACCCAGGGCAATgcgTTCCGCGACCA | pET28a-*depR* (C199A) |
| 3T11R | cgcATTGCCCTGGGTCAGCAGTAAGACGCT | pET28a-*depR* (C199A) |
| 3T12F | AATCTGCAAGGCAGCgCGCTCAACACCAT | pET28a-*depR* (S229A) |
| 3T12R | CGcGCTGCCTTGCAGATTGGCCGCCAGCG | pET28a-*depR* (S229A) |
| 3T13F | GGCAGCTCGCTCAACgCgATCCGCCACAT | pET28a-*depR* (T232A) |
| 3T13R | cGcGTTGAGCGAGCTGCCTTGCAGATTGG | pET28a-*depR* (T232A) |
| 3T14F | CAGCGGCATGGGCGTCgCGGTGATGCCGTC | pET28a-*depR* (T244A) |
| 3T14R | CGcGACGCCCATGCCGCTGGCCACCATGTG | pET28a-*depR* (T244A) |
| 3T15F | GACGCCGGCGCCGCAGgcgCGCGTGCTGTT | pET28a-*depR* (R271A) |
| 3T15R | cgcCTGCGGCGCCGGCGTCTTGAACGGCAC | pET28a-*depR* (R271A) |
| 3T16F | GTGCTGTTGGTGACTgcgAAGCAGTTCTTC | pET28a-*depR* (R278A) |
| 3T16R | cgcAGTCACCAACAGCACGCGGCGCTGCGG | pET28a-*depR* (R278A) |
| 3T17F | TGGCCTGCATTAGGCATTGTACTTG (EcoNI) | P*_orf22_* |
| 3T17R | TGGCCGCCATATGCATGACGACT (NdeI) | P*_orf22_* |
| DQ132F | CGATTCGCATATGCGTAA (NdeI) | *gfp* |
| DQ132R | ATCCTAGATCTTTATTTGTATAGTTCA (BglII) | *gfp* |
| DQ133F | GGGCATAAGTTGCGAGGCT | *depM* |
| DQ133R | TCTTCGATCAAGGGCACTCC | *depM* |
| DQ134F | GGTGTCGTGGTCACGCAGAC | *depA* |
| DQ134R | GCCATCAAATAGGCGGGAAAA | *depA* |
| DQ167F | GAACGGCTGCAAATCATCCT | *orf19*, RT-qPCR, 114bp |
| DQ167R | ATTGACCGCGACGTATTTGG | *orf19*, RT-qPCR, 114bp |
| DQ168R | GCGGCGCTGGCGGCCTT | *orf22* |
| XJ53F | CATGTTGACGGAACCTGCTGTGAT | Intergenic region of *depL-depM* |
| XJ53R | CATAGGCTTGTCCGCAGCTTTCTTA | Intergenic region of *depL-depM* |
| XJ54F | TAAGCAGCGGTTCAAAGTCCGCGC | Intergenic region of *depM-depN* |
| XJ54R | CATACGGGAAGAATGAAGCGTCAGCACT | Intergenic region of *depM-depN* |
| XJ55F | TGAGACACACTGTAAATCCTATTCTCT | Intergenic region of *depN-depA* |
| XJ55R | CATTGCAAGACCTCCATGTATCGACA | Intergenic region of *depN-depA* |
| XJ59F | GATCGTGGACCGTCCCGTGCGCGCC | Intergenic region of *orf20-orf21* |
| XJ59R | GTTTTACCGACGCCACCCTTGCCGG | Intergenic region of *orf20-orf21* |
| XJ69F | GGCGCCGGCCAGAACGATCGAGGA | Intergenic region of *orf21-orf22* |
| XJ69R | AGTCGGCTTGCCCAGAATGCTGTCG | Intergenic region of *orf21-orf22* |
| XJ69F-1 | GTCGACTGCGACTGGCCGAGAGCGG | Intergenic region of *orf21-orf22* |
| XJ69R-1 | CTCAGCTCATCCAGGTCGTCGGTGC | Intergenic region of *orf21-orf22* |
| XJ70F | GACTGCGACTGGCCGAGAGCGGA | Intergenic region of *orf21-orf22* R |
| XJ70R | AAATAGTGGTTTAGCGGCTTGTGCG | Intergenic region of *orf21-orf22* R |
| XJ71F | CGCACAAGCCGCTAAACCACTATTT | Intergenic region of *orf21-orf22* M |
| XJ71R | ATGGGGCGAGTGTACTGGGTTGCT | Intergenic region of *orf21-orf22* M |
| XJ72F | CATTGAAAGCCCGTCAGCAACCCAG | Intergenic region of *orf21-orf22* L |
| XJ72R-1 | GCTCATTGTACTTGGCTTTATGTAG | Intergenic region of *orf21-orf22* L |
| XJ75F | TTTTCCTAGAGGTCTTTCAGTATA | *depN* |
| XJ75R | TTTGAATCAAGAGAATAGGATTTA | *depN* |
| XJ79F | TGGCCTGCATTAGGCATGACGACT (EcoNI) | P*_orf21_* |
| XJ79R | TGGCCGCCATATGCATTGTACTTG (NdeI) | P*_orf21_* |
| XJ80F | TGGCCTGCATTAGGCATCCTGGCCTTAC (EcoNI) | P*_minC-Eco_* |
| XJ80R | TGGCCGCCATATGCATAGCACATCCTGA (NdeI) | P*_minC-Eco_* |
| YJ7F | CTGACCCAGGGCAATacCTTCCGCGAC | pET28a-*depR* (C199T) |
| YJ7R | gtATTGCCCTGGGTCAGCAGTAAGACG | pET28a-*depR* (C199T) |
| YJ9F | TCATGTTTTTGTCGGTCCAGTTCGC | *depN* |
| YJ9R | CCCATTCAAGAAGCAGTCATGCTGT | *depN* |
| YJ19R | GTAGCGAAACATCCACTGTAGTTGAC | Intergenic region of *depM-depN* L |
| YJ20F | TGGCTCAGGTTCTCTGCGAGGATTCG | Intergenic region of *depM-depN* R |
| YJ21F | TGTTCGCTTGCGGCGACATCACGC | Intergenic region of *depH-depI* |
| YJ21R | GCGCTCGCGCATTTCCTGAGCGGTC | Intergenic region of *depH-depI* |
| YJ22F | CGGCGTCGCGGTGGACTTCAAGATG | Intergenic region of *depI-depJ* |
| YJ22R | AAGCCGCTCCTCCCAGCCCCGGAAC | Intergenic region of *depI-depJ* |
| YJ39F | CAAGCCCGTTACGCTAGCCG | pYJ01 |
| YJ70F | CGTTTCTCCTCCATCGTGCT | *orf22*, RT-qPCR, 132 bp |
| YJ70R | GAAAGCCTTGGGATCCTTGG | *orf22*, RT-qPCR, 132 bp |
| YJ71F | GAGGCAGCAGTGGGGAATTT | 16S rDNA, RT-qPCR, 117 bp |
| YJ71R | AACCACTGGGATTTGCTCCC | 16S rDNA, RT-qPCR, 117 bp |
| YJ75F | CCGACGGCAACATCCATATC | *orf21*, RT-qPCR, 112 bp |
| YJ75R | GACACCAGTTCGGCTTCCAT | *orf21*, RT-qPCR, 112 bp |
| YJ80R | CCCAAGCTT TTATTTGTATAGTTCA (HindIII) | pWHU3064, pWHU3065 |
| YJ81F-1 | GAGGATATCCATGACGACTCCTAA (EcoRV) | pWHU3064 |
| YJ82F-1 | GAGGATATCCATTGTACTTG GCTT (EcoRV) | pWHU3065 |
| YJ85F | AGTCCATATGTTTACCACTACCCAAG (NdeI) | *orf22* |
| YJ85R | GACAAGCTTCGGAATCAGGCGGCGCT (HindIII) | *orf22* |

*Lowercase letters, codons of mutated amino acids
