# Supplementary figures and images for: Mechanistic studies of DepR in regulating FK228 biosynthesis in Chromobacterium violaceum no. 968
Source: PLoS One. 2018 Apr 19;13(4):e0196173. doi: 10.1371/journal.pone.0196173 (PMC5908139; doi:10.1371/journal.pone.0196173)

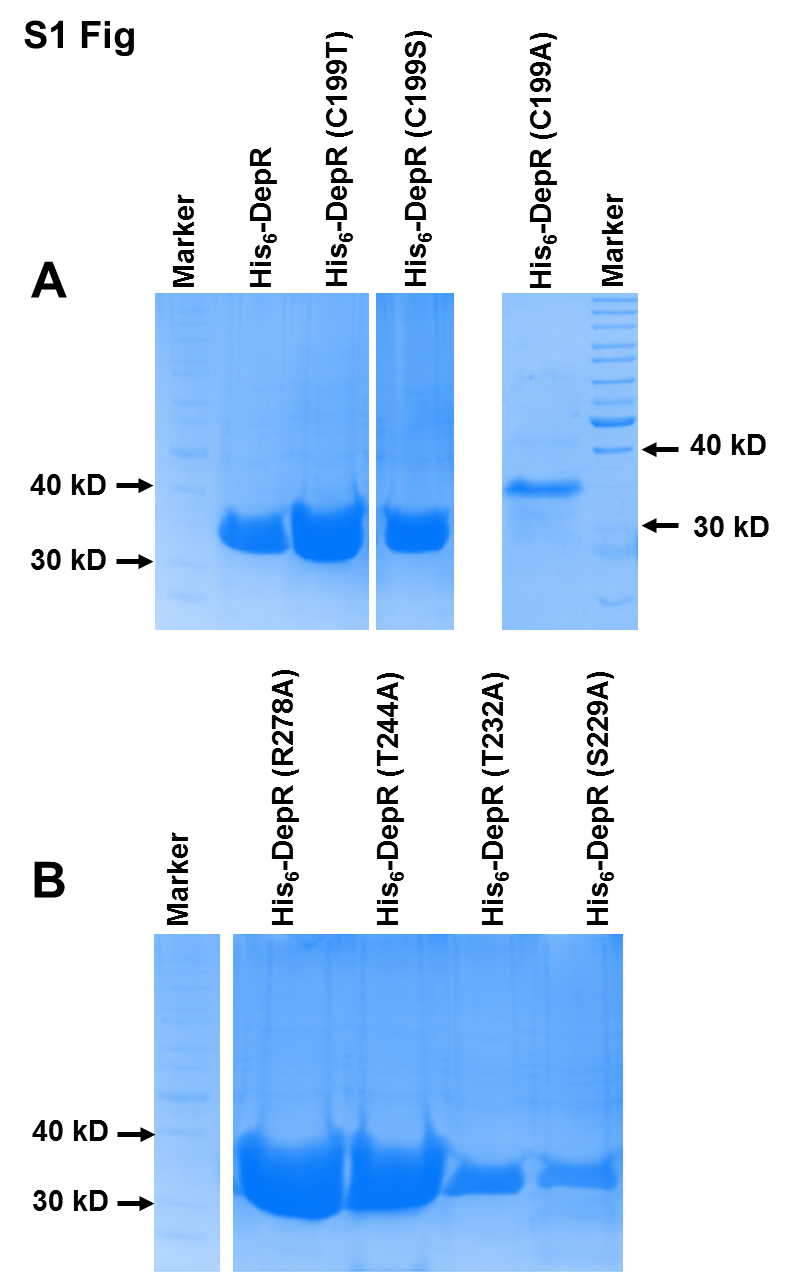

Supplement: S1 Fig — (TIF) [file pone.0196173.s003.tif]

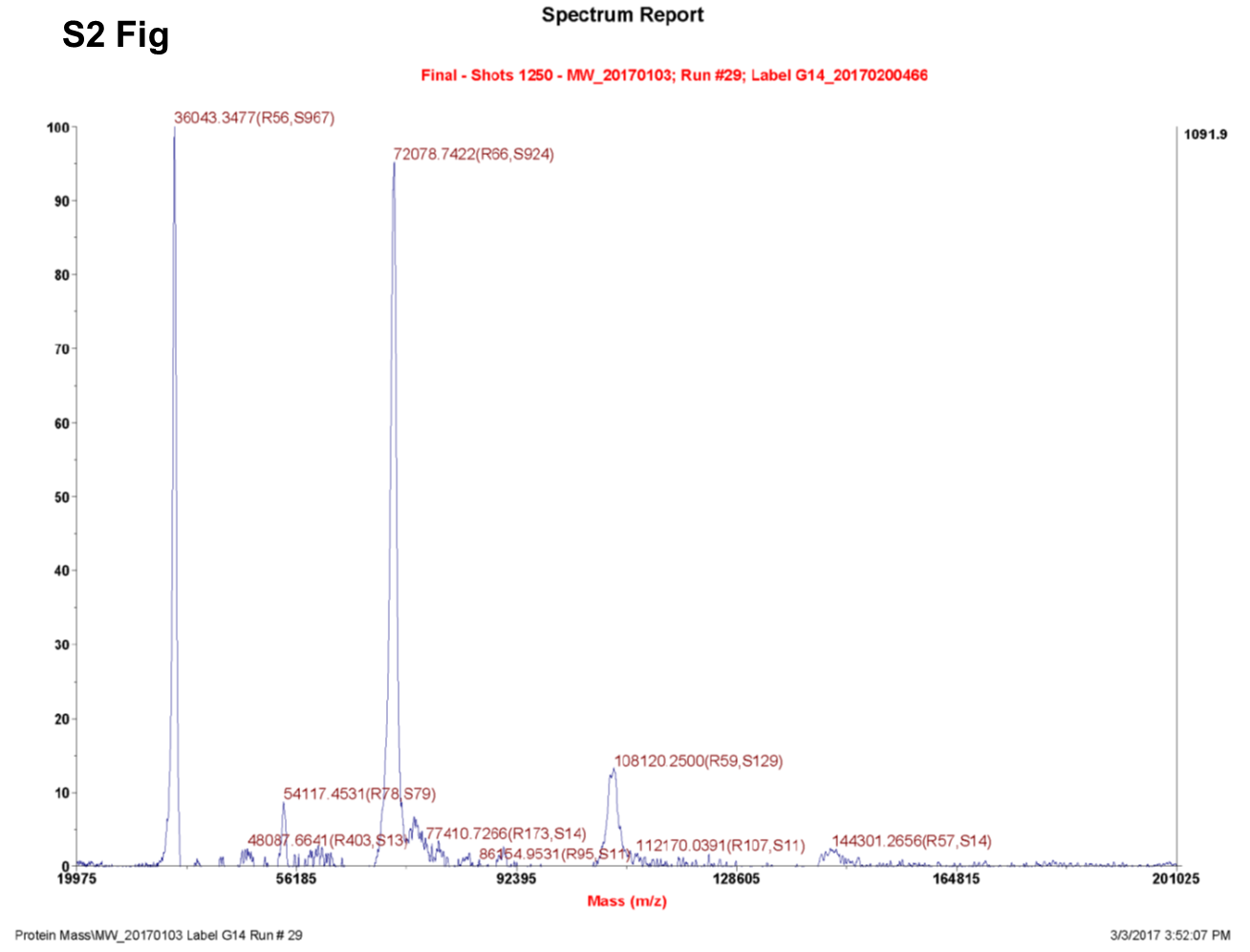

Supplement: S2 Fig — (TIF) [file pone.0196173.s004.tif]

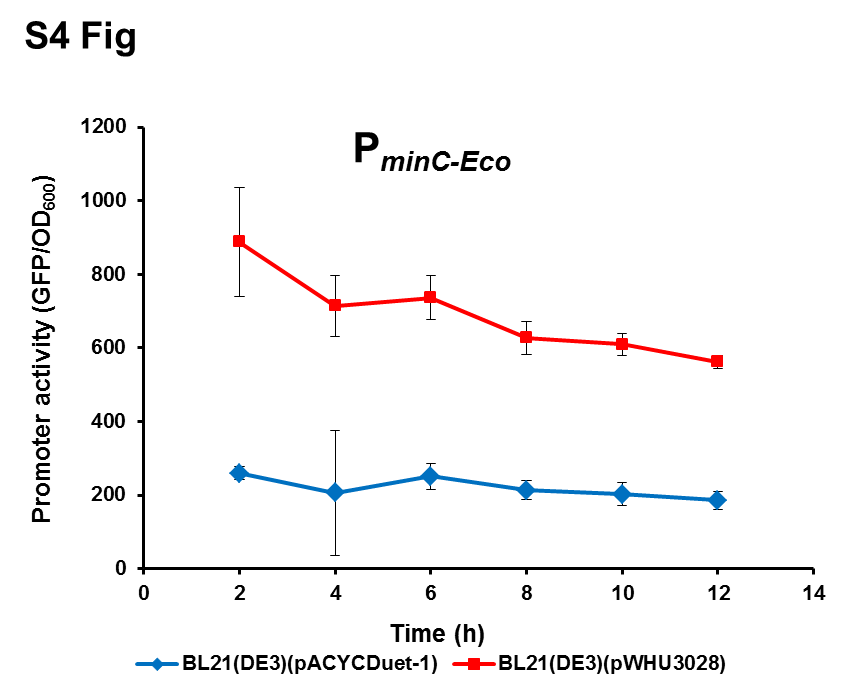

Supplement: S4 Fig — (TIF) [file pone.0196173.s006.tif]

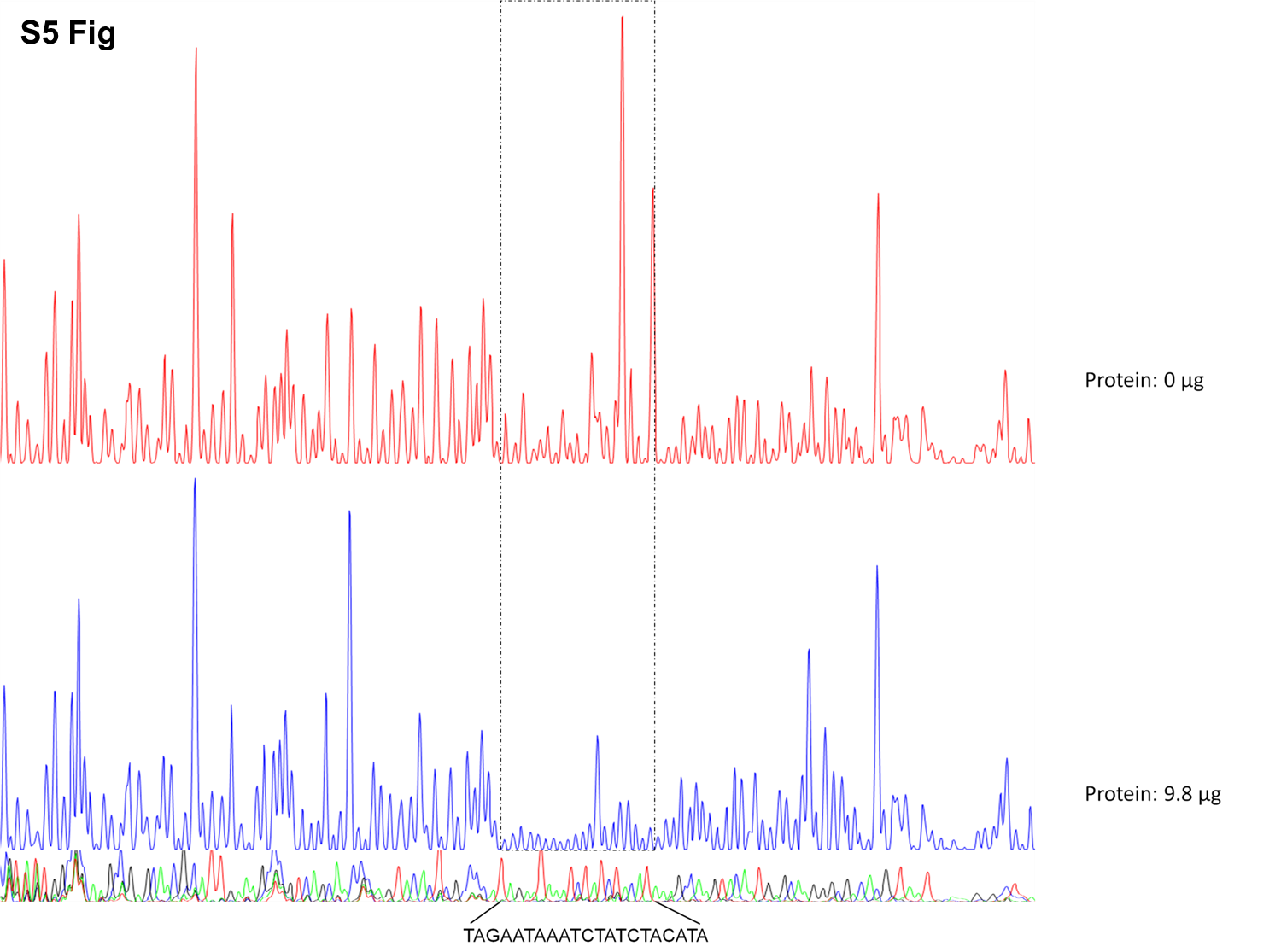

Supplement: S5 Fig — The upper two electropherograms indicate the reactions in the absence of protein and the presence of 9.8 μg (6.8 μM) purified protein DepR(C199S). The lower electropherogram represents the sequencing reaction. The DNA sequence (black) shows DepR (C199S) binding sites. (TIF) [file pone.0196173.s007.tif]

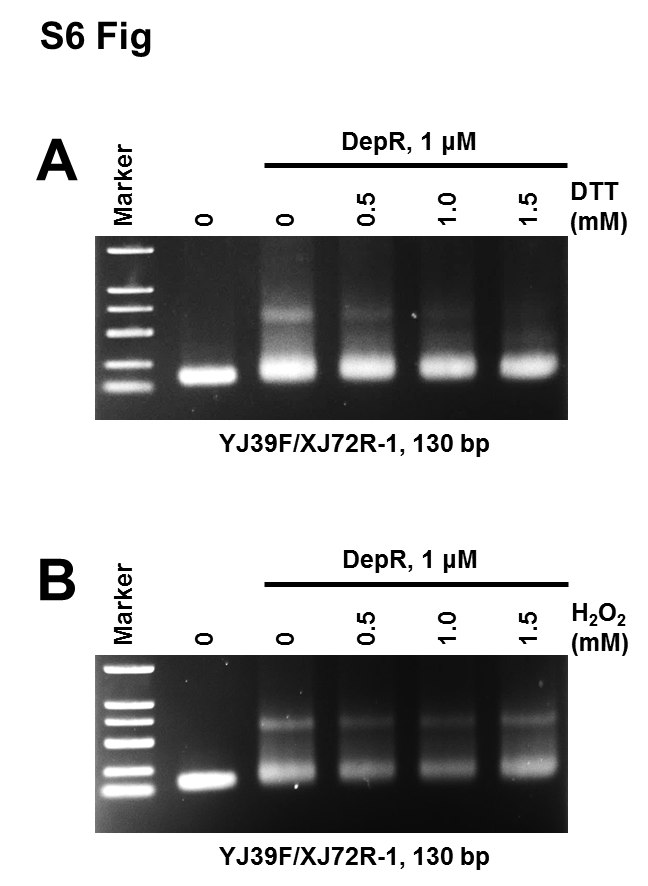

Supplement: S6 Fig — (A) EMSA analysis of DepR with target DNA fragments of DepR and 0–1.5 mM DTT. (B) EMSA analysis of DepR with target DNA fragments of DepR and 0–1.5 mM H2O2. (TIF) [file pone.0196173.s008.tif]

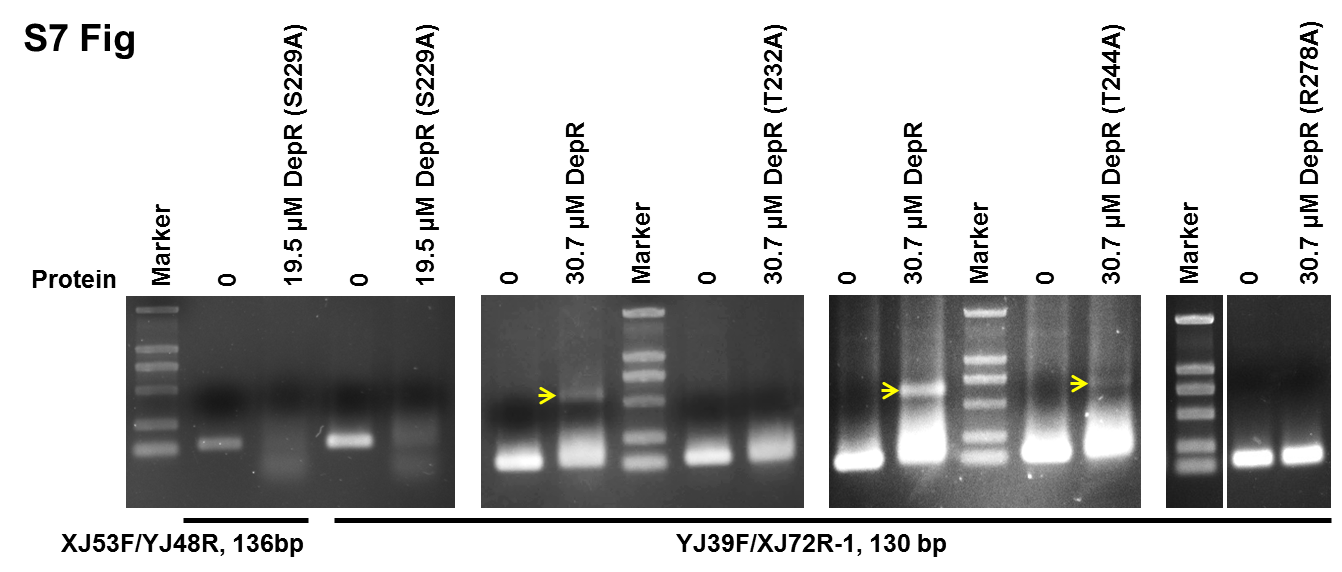

Supplement: S7 Fig — The bound DNA bands were indicated by yellow arrows. (TIF) [file pone.0196173.s009.tif]

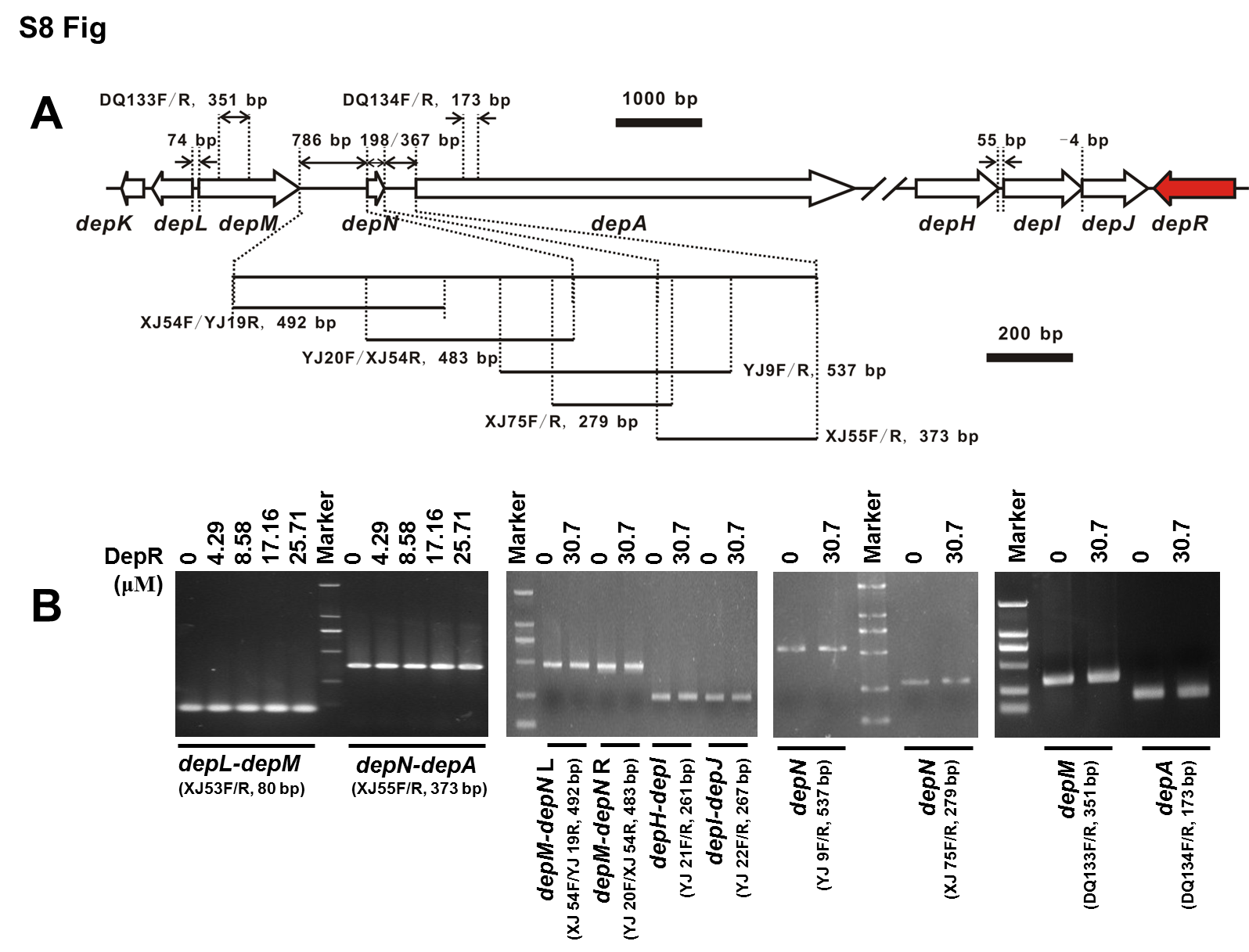

Supplement: S8 Fig — (A) dep gene cluster, primer pairs and the sizes and locations of DNA fragments amplified using in EMSA. (B) EMSA analysis of DepR with DNA fragments. (TIF) [file pone.0196173.s010.tif]
