# Supplementary material for: Mechanistic studies of DepR in regulating FK228 biosynthesis in Chromobacterium violaceum no. 968
Source: PLoS One. 2018 Apr 19;13(4):e0196173. doi: 10.1371/journal.pone.0196173 (PMC5908139; doi:10.1371/journal.pone.0196173)
Supplement: S3 Fig — The reverse complementary sequence of orf21 is marked in pink (previously published) and blue (determined in this work). The orf22 sequence (determined in this work) is marked in green. The start codons and stop codons of orf21 and orf22 are indicated by underlining. The orf21-orf22 intergenic region is marked in red. The 77-bp DNA region bound by DepR is shown in bold. The sequence has been deposited in GenBank with Accession Number MG696726. (PDF) [file pone.0196173.s005.pdf]

S3 Fig

5′-TTACTCGCCGAGCGCGGACATGACCAGGCGCTCGTTCTCGAGATAGATTTGAGT
CGGCTTGCCCAGAATGCTGTCGGGCAAGGCTTGTTCGATAGTTCGATAGACCCCGGC
GATGGACACCAGTTCGGCTTCCATGCTGCGGGCGAAAATGCGGGCGCCGGCGTTGCC
GCGGGCGCCGGCGAGAGCGCGGCCGCGCAAGGGCGCGTAGATATGGATGTTGCCGTC
GGCGATGACTTCAGCGCCGGCGCTGACCATGGCCAGCACGATCAGGTCGCAGCCTTT
GGCGTAAATCTGCTGACCGGCGCGCACGGGACGGTCCACGATCATGGCGCCGGTCGC
CTGGGCGGGTTCCGGCGCGGCGGGTTTGTCGGCAGGCTCCGGCGCGTTCTGCGCCGA
GCGCGGCGGCGTGGCGCTGCTGGTGTAGGCGAGGCCGAACTGGCTGGCGACGCCGGC
CAGCGCGGTGCTGGGGTGGCGCAGGGCCACCGCGCGGATGCCGCGGCGGCTCAGCAT
GGGCAGCAGGCGGCCGTAATCCAGTTCGGCCGGGGCGGGCAAGGCTTCCACGTCGAG
GACGAAGGCTTCTACCGGCGCGTCGTGGCCGGTGCCGAAGCGCGCATCCAGCGCCTG
GCTCAGCTCATCCAGGTCGTCGGTGCGCAGCAAGAGCGCCAGCAGATCCAGGCTGGC
GGATTTTATGTCGAATGCGTTGGCCGCAGG**GCTCATTGTACTTGGCTTTATGT**
AGATAGATTTATTCTATGGGGCGAGTGTACTGGGTTGCTGACGGGCTTT
CAATGGCGTTGCTCGGCTTGGGGCCAAAAGGTGGAAATCGGCTAGCGTAACGGGCT
TGGGCGCGGGCAAGTGTTGCAATTGCGGGAAACATCGCTAGAAGCGGGAGGGAAGGG
CCTTGTTGTCATGTCTCTGGAATGTTTGTGTTGTAAAAATCCATGAAAAATAGTGGT
TTAGCGGCTTGTGCGTTGCAACATCTTCTTGACTCGAGAAAGACGAGTCGCCACAAT
GGCAATTGTTGCGATGCAACATCAAGAGCGCAGGTTAAGTTCGCTGTCGTTGCTGAG
CTGTCAATTTTGGATATTTTTTAGGAGTCGTCATGTTTACCACTACCCAAGAATTCT
CCGCTCTCGGCCAGTCGCAGTTCGACAAGGCCGTGCGTTTCTCCTCCATCGTGCTGG
CCGGCGCTGAGCGTTTGGCCGCGTTGCAACTGGATCTGTCGCGCAAGCTGCTGGCAG
ACAACGCCCAGGCAATCAAGGCGCTGAGCGAAGCCAAGGATCCCAAGGCTTTCGCCG
ACGTGCAGAGCAGCCTGGCACAGCCGTCCATCGACCAGGCTTTCTCCGTGGCGCGCA
ATGTGTACGACGCCGCGCTGGCCACTCAGAATGAACTGGCCGCTTTTGTCGAAGAGC
AGATCGCCGAAGGCAACCAAACCCTGCTGAGCAATCTGGATCGTTTGTCCAAGAACG
CGCCGGCGGGCTCCGATGCCGCGGTGACGGCTCTGAAAAGCCTGGTGAACACTTCCA
ACGCCGCTTTCGAAAGCGTGTCCAAGACCGCCAAGAAAGTCAGCGCGGAAATCGCCG
AAGCCAGCGTTGAAGCCGCCACCAACTCCGCCAAGGCCGCCAGCGCCGCTGTCGCCC
GTGGCAAGAAGGCGACCAGCGCCGCCTGA-3′
